# The silent epidemic: exploring the link between loneliness and chronic diseases in China’s elderly

**DOI:** 10.1186/s12877-024-05163-2

**Published:** 2024-08-26

**Authors:** Lingbing Meng, Ruofan Xu, Jianyi Li, Jiabin Hu, Hongxuan Xu, Dishan Wu, Xing Hu, Xuezhai Zeng, Qiuxia Zhang, Juan Li, Deping Liu

**Affiliations:** 1 Department of Cardiology, Beijing Hospital, National Center of Gerontology, Institute of Geriatric Medicine, Chinese Academy of Medical Sciences, No. 1 DaHua Road, Dong Dan, Beijing, 100730 China; 2 Graduate School, Chinese Academy of Medical Sciences & Peking Union Medical College, No. 9 Dongdansantiao, Dongcheng District, Beijing, 100730 China; 3 Health Service Department of the Guard Bureau of the Joint Staff Department, Beijing, China; 4 China Research Center on Aging, Beijing, 100054 China; 5 Center on Aging Psychology, Key Laboratory of Mental Health, Institute of Psychology, Chinese Academy of Sciences, Beijing, China; 6 State Key Laboratory of Brain and Cognitive Science, Institute of Biophysics, Chinese Academy of Sciences, Beijing, China; 7 Department of Neurology, Beijing Hospital, National Center of Gerontology, Institute of Geriatric Medicine, Chinese Academy of Medical Sciences, No. 1 DaHua Road, Dong Dan, Beijing, 100730 China

**Keywords:** Loneliness, Aging, Anxiety, Endocrinology, Neuropsychiatry

## Abstract

**Background:**

Chronic diseases, such as heart disease, cancer, and diabetes, are the leading causes of death and disability. Loneliness is linked to a greater risk of chronic disease. However, the lack of loneliness may change this relationship.

**Methods:**

The 4th Survey of the Aged Population in Urban and Rural China (SSAPUR) was performed. 222,179 people over 60 years old were recruited. Chronic disease was defined by self-reported tumble incidents using the fourth SSAPUR questionnaire. We found that the residuals were well normally distributed. Subsequently, we analyzed the association between each studied factor and chronic disease by univariate logistic regression analysis. Finally, we stratified the population by age, gender, and urban and rural.

**Results:**

77,448 individuals experienced loneliness, while 137,593 did not. Loneliness correlated significantly with urban-rural classification, age, and gender (*P* < 0.001). There was a significant association between chronic diseases and loneliness (*P* < 0.05). Compared to lonely individuals, those with low level of loneliness had a lower incidence of gastric diseases (OR = 0.752, 95% CI, 0.736–0.769, *P* < 0.001), osteoarthritis (OR = 0.685, 95% CI, 0.673–0.697, *P* < 0.001), chronic obstructive pulmonary disease (COPD) (OR = 0.678, 95% CI, 0.659–0.698, *P* < 0.001), asthma (OR = 0.608, 95% CI, 0.583–0.633, *P* < 0.001), malignant tumors (OR = 0.892, 95% CI, 0.822–0.968, *P* = 0.006), and reproductive system diseases (OR = 0.871, 95% CI, 0.826–0.918, *P* < 0.001).

**Conclusion:**

In summary, loneliness is an important risk factor in the occurrence and development of chronic diseases in the elderly in China, and it has adverse effects on hypertension, stomach disease, cataract or glaucoma, osteoarthrosis, chronic lung disease, asthma, malignant tumor, and reproductive system diseases.

**Supplementary Information:**

The online version contains supplementary material available at 10.1186/s12877-024-05163-2.

## Introduction

Persistent health conditions are broadly characterized as ailments that extend for a duration of a year or more, necessitating consistent medical attention. These enduring conditions, exemplified by illnesses like heart disease, cancer, and diabetes, stand as the primary contributors to both loss of life and impaired functionality. According to data from the 2018 National Health Interview Survey (NHIS), over half (51.8%) of grown-ups grapple with a minimum of one among ten chronic conditions (namely arthritis, cancer, chronic obstructive pulmonary disease, coronary heart disease, asthma, diabetes, hepatitis, hypertension, stroke, and renal failure). Furthermore, a notable 27.2% of American adults are contending with multiple chronic ailments, highlighting the prevalence and impact of such enduring health challenges [1].

In the present era, a graceful shift towards a more mature global population is taking place. Between 2000 and 2020, the United States observed its most rapid demographic expansion within the serene age group of 65 years and beyond. Gazing into the future, a harmonious 56% increase is anticipated for individuals aged 60 or above by the year 2030. This phenomenon is not limited to any single nation; it encompasses the entire planet, painting a portrait of dignified aging. Envisioning the horizon of 2050, a symphony of growth unfolds as the worldwide elderly populace is poised to more than double. During this crescendo, the count of those aged 65 or older shall gracefully ascend to nearly 1.5 billion, with the majority finding their place within the nurturing embrace of developing nations [2]. China is embracing the challenges and opportunities that come with a maturing population. As of the end of 2015, there were approximately 222 million individuals aged 60 and older, making up about 16.1% of the total population. Among them, 143.86 million were in the distinguished age group of 65 and above, constituting around 10.5% of the total population. This demographic shift reflects the wisdom and experience of a significant population segment, contributing to the nation’s rich tapestry of life [3].

Among Chinese individuals aged over 60, the occurrence rates of hypertension, diabetes, and hypercholesterolemia stand at 58.3%, 19.4%, and 10.5%, respectively. It’s noteworthy that 75.8% of older adults contend with a minimum of one chronic ailment. Notably, the prevalence of chronic conditions escalates alongside age. Noteworthy chronic ailments affecting residents above 70 encompass stroke, myocardial infarction, cancer, and chronic obstructive pulmonary disease, constituting the most impactful health burdens [4]. These chronic conditions can precipitate mental and cognitive disorders, diminished mobility, cognitive decline, falls, and other concerns in the elderly population [5].

Loneliness is commonly understood as a psychological expression of social isolation. It mirrors an individual’s discontent with the regularity and closeness of their social engagements, in addition to the disparities between their existing relationships and their aspirations for more fulfilling connections [6]. Loneliness reflects a state of social isolation [7]. Loneliness is linked to various factors spanning socio-demographics, psychosocial elements, and health-related aspects. These encompass experiences like bereavement and divorce, as well as life circumstances like remaining unmarried and limited interactions with close friends. Furthermore, compromised physical health marked by chronic illnesses and reduced mobility, along with limited socio-economic resources including lower education and income levels, contribute to the presence of loneliness [8]. Research has indicated that the correlation between loneliness and blood pressure, as well as cardiovascular disease implies a cause-and-effect relationship rooted in physiological mechanisms [7, 9]. Sugisawa et al. discovered a notable impact of loneliness on mortality within a three-year timeframe. This influence can be attributed to chronic illnesses, functional capabilities, and self-perceived health conditions [10]. During a 19-year follow-up period in the National Health and Nutrition Survey, it was observed that persistent high-frequency loneliness (occurring more than 3 days a week at intervals of approximately 8 years in both assessments) among women was linked to an increased risk of coronary heart disease (CHD). This association remained significant even after accounting for factors such as age, race, socioeconomic status, marital status, and other cardiovascular risk factors [11]. Loneliness is linked to a greater risk of peptic ulcer recurrence. However, the absence of or decreased severity of loneliness may change this relationship [12].

Hence, the 4th Survey of the Elderly Population in Urban and Rural China (SSAPUR) was conducted to examine the correlation between loneliness and persistent stress. The primary objective of this research is to uncover the impact of loneliness on chronic ailments by delving into societal, psychological, and behavioral dimensions. The ultimate aim is to offer empirical data that can contribute to the development of guidelines for preventing and treating chronic conditions among elderly individuals, both in China and globally.

## Methods

### Basic information

#### Research project

The 4th Survey of the Aged Population in Urban and Rural China (SSAPUR). The complete long form version of the SSAPUR survey questionnaire that we have based our study on was presented in Supplementary Materials. To standardize the procedure of project, a directory of investigator’s manual was designed and supplied to visit in Supplementary Materials.

#### Research population and basic characteristics

This national cross-sectional study encompassed a total of 222,179 individuals aged 60 and above, representing every province, autonomous region, centrally-administered municipality, as well as the Xinjiang Production and Construction Corps. The research spanned 466 counties (districts), 1864 townships (streets), and 7456 village (neighborhood) committees, resulting in 219,404 eligible and valid questionnaires collected.

#### Survey content

Our investigation centers on health-related data and delves into risk factors associated with loneliness and chronic illnesses. This research encompasses a wide array of factors including population, economic conditions, healthcare services, societal engagement, cultural aspects, and living environments, among others.

### Study protocol

This study utilized data from the 2015 iteration of the fourth SSAPUR survey, which stands as the most extensive dataset available for elderly individuals. Employing a multilevel hierarchical cluster sampling approach, the fourth survey incorporated equal probability sampling at its final level. Comprehensive details regarding the survey’s design and sampling methods are documented in a prior publication of ours. This survey marked a significant milestone by successfully attaining national-scale objectives, offering insights into the socioeconomic status and lifestyles of older adults in China. The research protocol secured approval from both the Ethical Review Committee of Beijing Hospital (2021BJYYEC-294-01) and the National Bureau of Statistics (No. [2014] 87). Prior to participation, all respondents provided written informed consent.

### Questionnaire

We used the fourth SSAPUR questionnaire, which has been used in large-scale epidemiological studies, including the global burden of disease study and the World Health Survey. Urban and rural, age, sex, education level, marital status, whether living alone, smoking or not, drinking or not, sleep status, exercise status, whether there is medical insurance, whether there are other elderly people in the home who need care, whether they are engaged in paid work, economic status, whether they participate in public welfare activities, whether they have external abuse, and whether they participate in a spiritual-cultural life.

### Diseases, loneliness, and older age group definitions

We categorized chronic diseases and loneliness based on self-reported occurrences of falls, as outlined in the fourth SSAPUR questionnaire. Chronic diseases include glaucoma/cataracts, hypertension, diabetes, gastric diseases, cardiovascular disease, osteoarthritis, chronic lung disease, asthma, cancer, and reproductive system disorders. The evaluation of loneliness was also based on the SSAPUR questionnaire and the measurement of loneliness depended on the responses of the participants regarding their own loneliness situation, and it is divided into two levels (Often = 1, never = 0). Questionnaire’s validity has been confirmed through rigorous validation procedures and its application in numerous regional surveys across China. Additionally, we validated its effectiveness through an internal study. Furthermore, in accordance with both international standards and Chinese legislation, individuals aged 60 and above were considered elderly for the purpose of our study.

### Statistical analysis

We studied the Chinese elderly population aged 60 and older, stratified by sampling results, based on the above questionnaire results. The age-standardized prevalence of tumble was calculated from the sampling results. We excluded 7121 respondents with unclear tumble status from 222,179 survey data and 17 respondents with more than ten missing data on independent variables. As a result, 219,404 data was included in our analysis.

During the course of multiple group comparisons, our initial step involved assessing the statistical significance of distinctions among various groups using the χ²-test for categorical variables. Subsequently, we employed a two-sided Newman-Keuls test for post hoc analysis. We then verified residual distributions through histograms and the Shapiro-Wilk test, followed by employing the Wilcoxon signed rank test for comparing non-normally distributed data. Notably, our examination revealed the satisfactory normal distribution of residuals.

In the subsequent stage, we conducted univariate logistic regression analyses to explore the link between each examined factor and chronic diseases. Further stratification of the population was executed based on age, gender, and urban or rural classification, with *P* values below 0.05 considered as indicators of statistical significance. All statistical computations were conducted using SPSS 24.0 (IBM Corp., Armonk, NY, USA).

## Results

### Baseline information of participants based on different levels of loneliness

**Fig. 1:**
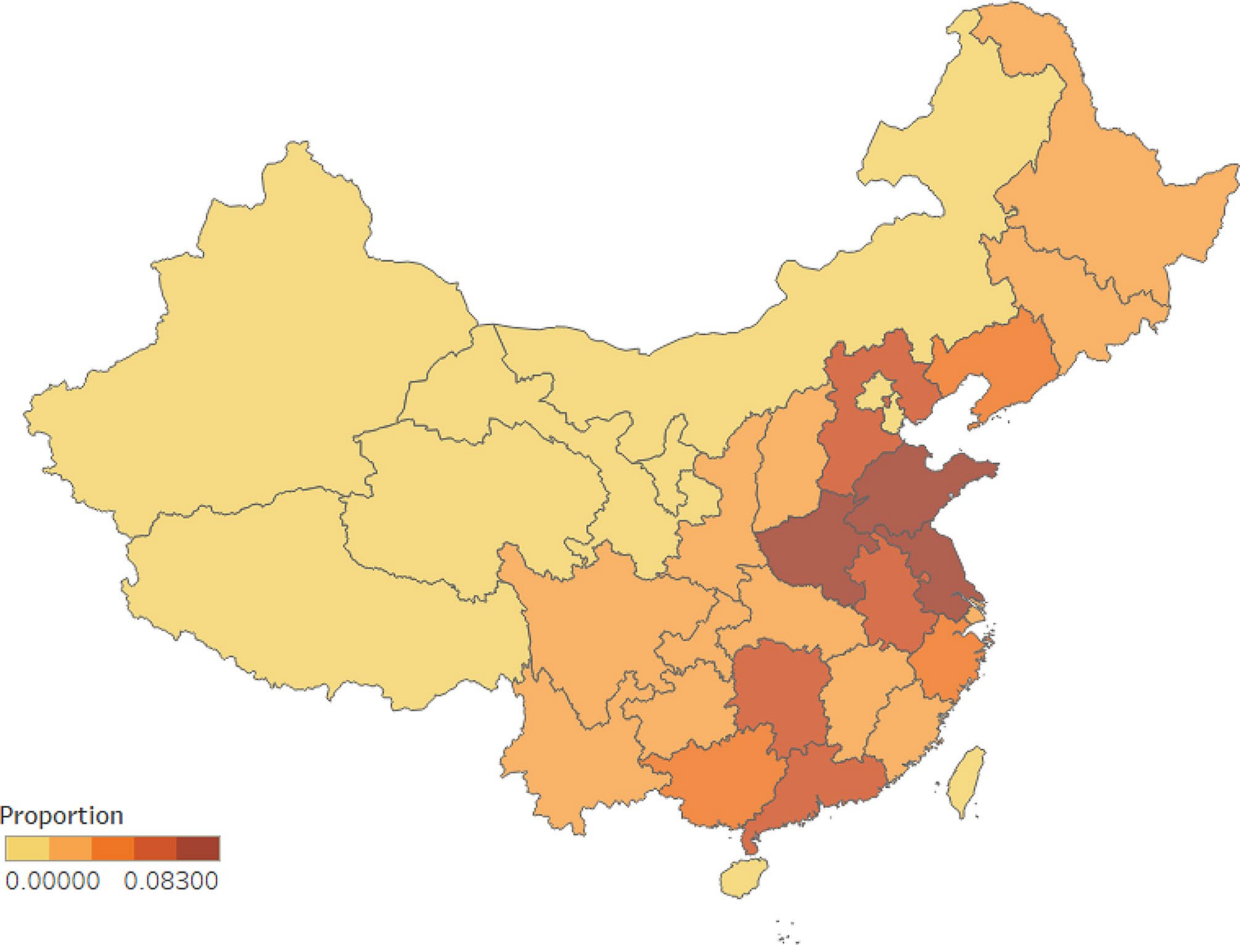
The proportion of investigators in different provinces

**Fig. 2:**
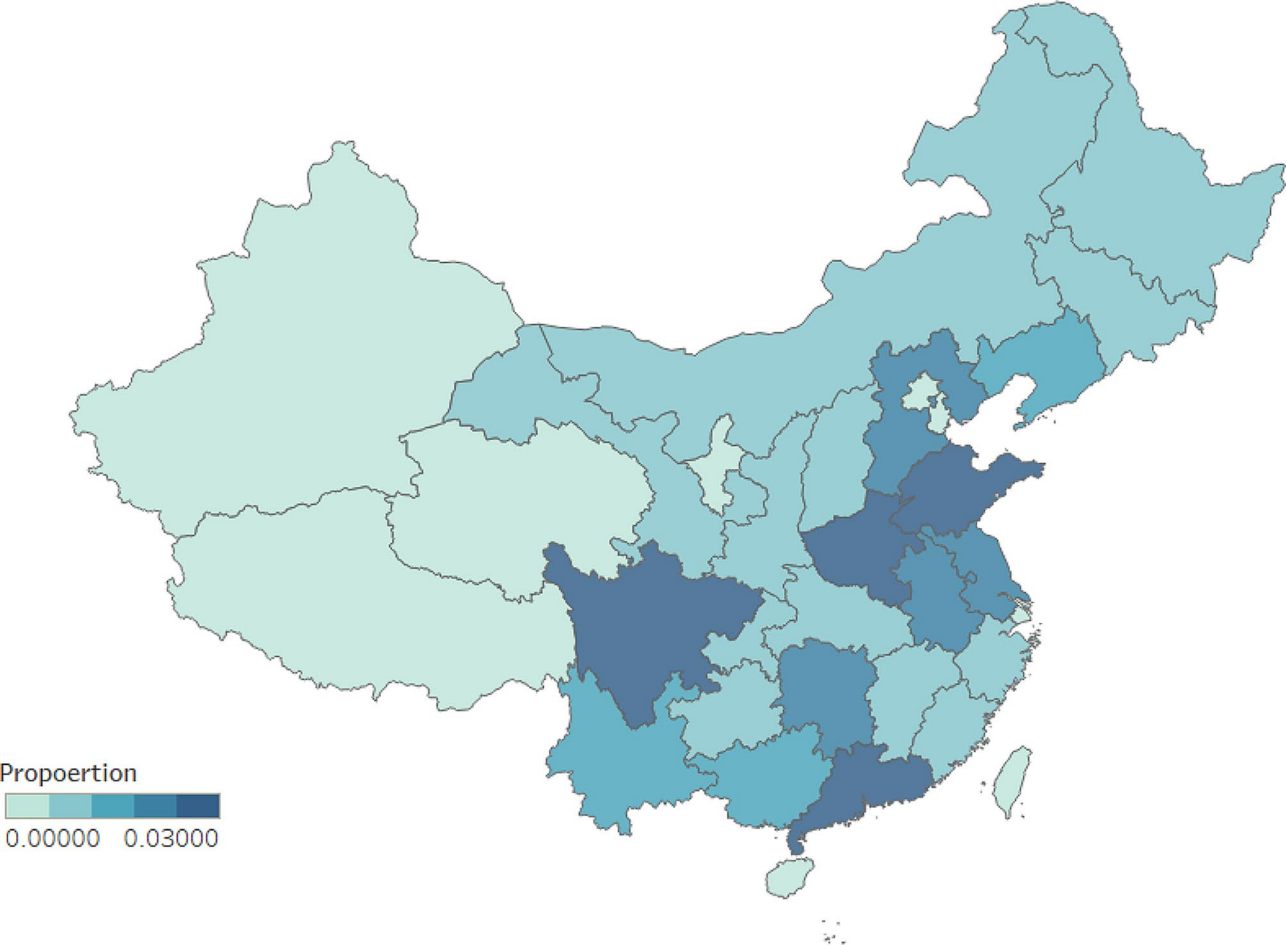
Prevalence of the loneliness in each province in the China

The proportion of investigators in different provinces is presented in Fig. 1. And the prevalence of loneliness in each province in China is manifested in Fig. 2. There was a total of 111,940 participants from the town and 103,101 individuals from the rural area. And 77,448 people were with loneliness, and 137,593 individuals were in low level of loneliness. Loneliness was significantly related with the “urban and rural”, “age”, and “sex” (*P* < 0.001). Chronic diseases include hypertension, diabetes, gastropathy, cataract and glaucoma, osteoarthropathy, chronic obstructive pulmonary disease, asthma, malignancy, and reproductive system disease. And the above chronic diseases were obviously related to loneliness (*P* < 0.05). (Table 1)

**Table 1:** The based information of participants with (or without) loneliness

Factor	Total (proportion)	Loneliness	Low level of loneliness	
				*P*-value
Urban and rural				< 0.001
Town	111,940 (0.521)	33,091(0.154)	78,849(0.367)	
Rural area	103,101 (0.479)	44,357(0.206)	58,744(0.273)	
Age(years)				< 0.001
60–64	70,913(0.33)	19,705(0.304)	51,208(0.026)	
65–69	50,723(0.236)	16,515(0.077)	34,208(0.159)	
70–74	35,760(0.166)	13,572(0.063)	22,188(0.103)	
75–79	28,131(0.131)	12,305(0.057)	15,826(0.074)	
80–84	18,426(0.086)	9078(0.042)	9348(0.043)	
≥ 85	11,088(0.052)	6273(0.029)	4815(0.022)	
Sex				< 0.001
Female	112,349(0.522)	44,112(0.205)	68,237(0.317)	
Male	102,692(0.478)	33,336(0.155)	69,356(0.323)	
Hypertension				< 0.001
No	135,768(0.631)	47,756(0.222)	88,012(0.409)	
Yes	79,273(0.369)	29,692(01.38)	49,581(0.231)	
Diabetes				< 0.001
No	196,319(0.913)	71,013(0.33)	125,306(0.583)	
Yes	18,722(0.087)	6435(0.03)	12,287(0.057)	
Gastropathy				< 0.001
No	176,690(0.822)	61,523(0.286)	115,167(0.536)	
Yes	38,351(0.178)	15,925(0.074)	22,426(0.104)	
Cataract and Glaucoma				< 0.001
No	180,626(0.84)	63,040(0.293)	117,586(0.547)	
Yes	34,415(0.16)	14,408(0.067)	20,007(0.093)	
Osteoarthropathy				< 0.001
No	121,140(0.563)	38,996(0.181)	82,144(0.382)	
Yes	93,901(0.437)	38,452(0.179)	55,449(0.258)	
COPD (chronic obstructive pulmonary disease)				< 0.001
No	193,092(0.898)	67,718(0.315)	125,374(0.583)	
Yes	21,949(0.102)	9730(0.045)	12,219(0.057)	
Asthma				< 0.001
No	205,405(0.955)	72,871(0.339)	132,534(0.616)	
Yes	9636(0.045)	4577(0.021)	5059(0.024)	
Malignancy				0.007
No	212,578(0.989)	76,496(0.356)	136,082(0.633)	
Yes	2463(0.011)	952(0.004)	1511(0.007)	
Reproductive System Disease				< 0.001
No	209,078(0.972)	75,112(0.349)	133,966(0.623)	
Yes	5963(0.028)	2336(0.011)	3627(0.017)	

### Correlation analysis of chronic disease to loneliness

The correlation analysis was conducted between the low level loneliness and chronic diseases. Low level of loneliness showed negative correlation with the “urban and rural” (*r*=-0.140, *P* < 0.001), “age” (*r*=-0.168, *P* < 0.001), “hypertension” (*r*=-0.023, *P* < 0.001), “gastropathy” (*r*=-0.053, *P* < 0.001), “cardiovascular disease” (*r*=-0.016, *P* < 0.001), “Cataract and Glaucoma” (*r*=-0.053, *P* < 0.001), “Osteoarthropathy” (*r*=-0.090, *P* < 0.001), “chronic obstructive pulmonary disease” (*r*=-0.058, *P* < 0.001), “asthma” (*P* < 0.001), and the Malignancy (*r*=-0.006, *P* = 0.006), Reproductive System Diseases (*r*=-0.011, *P* < 0.001) (Table 2). Alternatively, low level of loneliness exhibited positive correlation with “sex” (*r* = 0.071, *P* < 0.001) and “diabetes” (*r* = 0.011, *P* < 0.001).

**Table 2:** Correlation analysis of other related factors to loneliness

Factors	Low level of Loneliness		
	Correlation	*p*-value	
Urban and rural	-0.140	< 0.001	
Age	-0.168	< 0.001	
Sex	0.071	< 0.001	
Hypertension	-0.023	< 0.001	
Diabetes	0.011	< 0.001	
Gastropathy	-0.053	< 0.001	
Cardiovascular disease	-0.016	< 0.001	
Cataract and Glaucoma	-0.053	< 0.001	
Osteoarthropathy	-0.090	< 0.001	
chronic obstructive pulmonary disease	-0.058	< 0.001	
Asthma	-0.052	< 0.001	
Malignancy	-0.006	0.006	
Reproductive System Diseases	-0.011	< 0.001	

### Effect of loneliness on chronic disease based on the univariate analysis

**Fig. 3:**
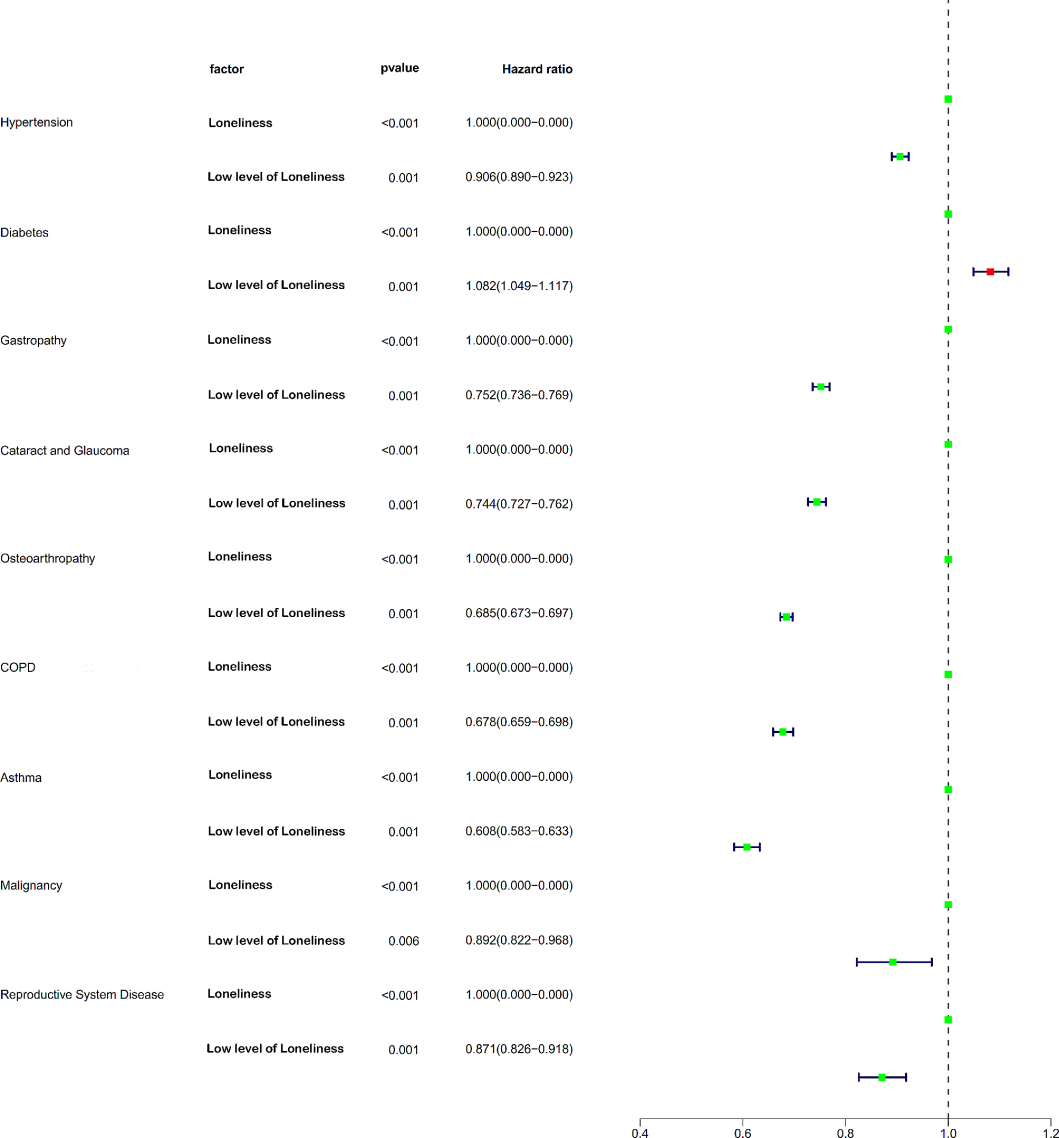
Effect of loneliness on the chronic disease based on the univariate analysis

Individuals with low level of loneliness had lower incidence rates of various health conditions compared to those with loneliness. The odds ratios (OR) for those with low level of loneliness were significantly lower for hypertension (OR = 0.906), gastropathy (OR = 0.752), cataract and glaucoma (OR = 0.744), osteoarthropathy (OR = 0.685), chronic obstructive pulmonary disease (OR = 0.678), asthma (OR = 0.608), malignancy (OR = 0.892), and reproductive system disease (OR = 0.871), with all *p*-values being less than 0.001 except for malignancy (*p* = 0.006). However, the OR for those with low level of loneliness in diabetes was (OR = 1.082, *P* < 0.001). (Table 3; Fig. 3)

**Table 3:** Univariate analysis for loneliness

Factor	Univariate	
	OR(95%CI)	*P*-value
Hypertension		
With loneliness	1	
Low level of loneliness	0.906(0.890–0.923)	< 0.001
Diabetes		
With loneliness	1	
Low level of loneliness	1.082(1.049–1.117)	< 0.001
Gastropathy		<
With loneliness	1	
Low level of loneliness	0.752(0.736–0.769)	< 0.001
Cataract and Glaucoma		
With loneliness	1	
Low level of loneliness	0.744(0.727–0.762)	< 0.001
Osteoarthropathy		
With loneliness	1	
Low level of loneliness	0.685(0.673–0.697)	< 0.001
COPD(chronic obstructive pulmonary disease)		
With loneliness	1	
Low level of loneliness	0.678(0.659–0.698)	< 0.001
Asthma		< 0.001
With loneliness	1	
Low level of loneliness	0.608(0.583–0.633)	< 0.001
Malignancy		< 0.001
With loneliness	1	
Low level of loneliness	0.892(0.822–0.968)	0.006
Reproductive System Disease		< 0.001
With loneliness	1	
Low level of loneliness	0.871(0.826–0.918)	< 0.001

### Stratification analysis based on the age stage

Based on age, the participants were divided into three groups, including 60–69, 70–79, and ≥ 80 years old. In the age stage “60–69”, loneliness was correlated with the hypertension (*P* < 0.001). However, in the other age stage, loneliness was not correlated with hypertension (*P* > 0.05). In all the different stages of age, loneliness was related to Gastropathy, Cataracts and Glaucoma, Osteoarthropathy, COPD, Asthma, and Reproductive System Diseases (*P* < 0.001). In the age stage “60–69”, loneliness was correlated with the malignancy (*P* < 0.001). However, in the other age stage, loneliness was not correlated with malignancy (*P* > 0.05). (Table 4)

**Table 4:** The analysis for the relationship between parameters and loneliness by “Age” stratification

Factor	All			Age								
				60–69			70–79			>=80		
	With Loneliness	Low level of loneliness	*P*-value	With Loneliness	Low level of loneliness	*P*-value	With Loneliness	Low level of loneliness	*P*-value	With Loneliness	Low level of loneliness	*P*-value
Sex			< 0.001			< 0.001			< 0.001			< 0.001
Female	44,112	68,237		19,571	42,297		14,918	18,537		9623	7403	
Male	33,336	69,356		16,649	43,119		10,959	19,477		5728	6760	
Urban and rural			< 0.001			< 0.001			< 0.001			< 0.001
Town	33,091	78,849		14,855	47,191		10,996	22,462		7240	9196	
Rural area	44,357	58,744		21,365	38,225		14,881	15,552		8111	4967	
Hypertension			< 0.001			< 0.001			0.053			0.719
No	47,756	88,012		23,659	57,240		15,037	22,383		9060	8389	
Yes	29,692	49,581		12,561	28,176		10,840	15,631		6291	5774	
Diabetes			< 0.001			0.204			< 0.001			< 0.001
No	71,013	125,306		33,234	78,184		23,578	34,224		14,201	12,898	
Yes	6435	12,287		2986	7232		2299	3790		1150	1265	
Gastropathy			< 0.001			< 0.001			< 0.001			< 0.001
No	61,523	115,167		27,974	70,796		20,661	32,014		12,888	12,357	
Yes	15,925	22,426		8246	14,620		5216	6000		2463	1806	
Cataracts and Glaucoma			< 0.001			< 0.001			< 0.001			< 0.001
No	63,040	117,586		31,530	76,496		20,431	30,604		11,079	10,486	
Yes	14,408	20,007		4690	8920		5446	7410		4272	3677	
Osteoarthropathy			< 0.001			< 0.001			< 0.001			< 0.001
No	38,996	82,144		18,544	51,655		12,688	22,040		7764	8449	
Yes	38,452	55,449		17,676	33,761		13,189	15,974		7587	5714	
COPD			< 0.001			< 0.001			< 0.001			< 0.001
No	67,718	125,374		32,182	79,077		22,314	33,864		13,222	12,433	
Yes	9730	12,219		4038	6339		3563	4150		2129	1730	
Asthma			< 0.001			< 0.001			< 0.001			< 0.001
No	72,871	132,534		34,459	82,901		24,207	36,233		14,205	13,400	
Yes	4577	5059		1761	2515		1670	1781		1146	763	
Malignancy			0.007			< 0.001			0.480			0.201
No	76,496	136,082		35,719	84,491		25,560	37,573		15,217	14,018	
Yes	952	1511		501	925		317	441		134	145	
Reproductive System Diseases			< 0.001			< 0.001			0.034			< 0.001
No	75,112	133,966		35,194	83,652		25,097	36,753		14,821	13,561	
Yes	2336	3627		1025	1764		780	1261		530	602	

### Stratification analysis based on the gender or the urban and rural

In the female population, loneliness was correlated with hypertension (*P* < 0.001). However, in the male population, loneliness was not correlated with the hypertension (*P* = 0.131). In the male population, loneliness was associated with the diabetes (*P* < 0.001). By contrast, in the female population, loneliness was not correlated with the diabetes (*P* = 0.06). In the male population, loneliness was correlated with malignancy (*P* = 0.002). Interestingly, in the female population, loneliness was not correlated with malignancy (*P* = 0.348).(Table 5) In the “urban and rural” stratification analysis, loneliness was associated to diabetes in all sample (*P* < 0.001) while showed no significance in urban and rural group. (Table 6)

**Table 5:** The analysis for the relationship between parameters and loneliness by gender stratification

Factor	All			Female			Male		
	With Loneliness	Low level of loneliness	*P*-value	With Loneliness	Low level of loneliness	*P*-value	With Loneliness	Low level of loneliness	*P*-value
Urban and rural			< 0.001			< 0.001			< 0.001
Town	33,091	78,849		19,894	39,776		13,197	39,073	
Rural area	44,357	58,744		24,218	28,461		20,139	30,283	
Age(years)			< 0.001			< 0.001			< 0.001
60–64	19,705	51,208		10,616	25,507		9089	25,701	
65–69	16,515	34,208		8955	16,790		7560	17,418	
70–74	13,572	22,188		7706	10,778		5866	11,410	
75–79	12,305	15,826		7212	7759		5093	8067	
80–84	9078	9348		5595	4723		3483	4625	
≥ 85	6273	4815		4028	2680		2245	2135	
Hypertension			< 0.001			< 0.001			0.131
No	47,756	88,012		25,845	42,094		21,911	45,918	
Yes	29,692	49,581		18,267	26,143		11,425	23,438	
Diabetes			< 0.001			0.06			< 0.001
No	71,013	125,306		39,899	61,486		31,114	63,820	
Yes	6435	12,287		4213	6751		2222	5536	
Gastropathy			< 0.001			< 0.001			< 0.001
No	61,523	115,167		34,303	56,058		27,220	59,109	
Yes	15,925	22,426		9809	12,179		6116	10,247	
Cataract and Glaucoma			< 0.001			< 0.001			< 0.001
No	63,040	117,586		34,471	56,396		28,569	61,190	
Yes	14,408	20,007		9641	11,841		4767	8166	
Osteoarthropathy			< 0.001			< 0.001			< 0.001
No	38,996	82,144		20,210	36,817		18,786	45,327	
Yes	38,452	55,449		23,902	31,420		14,550	24,029	
COPD (chronic obstructive pulmonary disease)			< 0.001			< 0.001			< 0.001
No	67,718	125,374		39,306	63,159		28,412	62,215	
Yes	9730	12,219		4806	5078		4924	7141	
Asthma			< 0.001			< 0.001			< 0.001
No	72,871	132,534		41,658	65,923		31,213	66,611	
Yes	4577	5059		2454	2314		2123	2745	
Malignancy			0.007			0.348			0.002
No	76,496	136,082		43,605	67,495		32,891	68,587	
Yes	952	1511		507	742		445	769	
Reproductive System Disease			< 0.001			< 0.001			< 0.001
No	75,112	133,966		43,219	67,187		31,893	66,779	
Yes	2336	3627		893	1050		1443	2577	

**Table 6:** The analysis for the relationship between loneliness by “urban and rural” stratification

Factor	All			Urban			Rural		
	With loneliness	Low level of loneliness	*P*-value	With loneliness	Low level of loneliness	*P*-value	With loneliness	Low level of loneliness	*P*-value
Age(years)			< 0.001			< 0.001			< 0.001
60–64	19,705	51,208		8055	28,114		11,650	23,094	
65–69	16,515	34,208		6800	19,077		9715	15,131	
70–74	13,572	22,188		5626	12,914		7946	9274	
75–79	12,305	15,826		5370	9548		6935	6278	
80–84	9078	9348		4261	6105		4817	3243	
≥ 85	6273	4815		2979	3091		3294	1724	
Sex			< 0.001			< 0.001			< 0.001
Female	44,112	68,237		19,894	39,776		24,218	28,461	
Male	33,336	69,356		13,197	39,073		20,139	30,283	
Hypertension			< 0.001			< 0.001			< 0.001
No	47,756	88,012		19,153	48,147		28,603	39,865	
Yes	29,692	49,581		13,938	30,702		15,754	18,879	
Diabetes			< 0.001			0.339			0.211
No	71,013	125,306		29,281	69,929		41,732	55,377	
Yes	6435	12,287		3810	8920		2625	3367	
Gastropathy			< 0.001			< 0.001			< 0.001
No	61,523	115,167		27,056	67,822		34,467	47,345	
Yes	15,925	22,426		6035	11,027		9890	11,399	
Cataract and Glaucoma			< 0.001			< 0.001			< 0.001
No	63,040	117,586		26,104	66,150		36,936	51,436	
Yes	14,408	20,007		6987	12,699		7421	7308	
osteoarthropathy			< 0.001			< 0.001			< 0.001
No	38,996	82,144		17,487	49,943		21,509	32,201	
Yes	38,452	55,449		15,604	28,906		22,848	26,543	
COPD (chronic obstructive pulmonary disease)			< 0.001			< 0.001			< 0.001
No	67,718	125,374		29,382	72,539		38,336	52,835	
Yes	9730	12,219		3709	6310		6021	5909	
Asthma			< 0.001			< 0.001			< 0.001
No	72,871	132,634		31,492	76,425		41,379	56,109	
Yes	4577	5059		1599	2424		2978	2635	
Malignancy			0.007			0.001			0.007
No	76,496	136,082		32,570	77,815		43,026	58,267	
Yes	952	1511		521	1034		431	477	
Reproductive System Disease			< 0.001			< 0.001			< 0.001
No	75,112	133,966		32,014	76,647		43,098	57,319	
Yes	2336	3627		1077	2202		1259	1425	

## Discussion

In Chinese individuals aged 60, rates of hypertension, diabetes, and hypercholesterolemia were 58.3%, 19.4%, and 10.5% respectively. Up to 75.8% suffered from at least one chronic disease, with higher prevalence in women and urban areas. Furthermore, a noticeable trend emerged with advancing age, wherein the prevalence of chronic diseases escalated. Among residents aged 70 and above, the most burdensome chronic conditions were identified as stroke, myocardial infarction, cancer, and chronic obstructive pulmonary disease (COPD) [4].

Studies show increased chronic diseases among the elderly. 14.5% have hypertension, 6.4% have both high blood pressure and diabetes, 3.8% have heart disease and hypertension, 3.2% have hypertension and osteoarthritis, and 1.6% have hypertension with COPD. Obviously, chronic diseases cause significant annual medical costs [13, 14].

Loneliness has been linked to cellular functional alterations and heightened vascular resistance [15], while also correlating with a higher likelihood of several specific conditions. These conditions encompass depression, cognitive decline, and the advancement of Alzheimer’s disease [16], as well as obesity [17], stroke, and hypertension [18]. The main reason is that loneliness can trigger stress responses, increase blood pressure, and weaken the immune system due to hormonal and inflammatory changes [16, 19, 20]. By contrast, some chronic illnesses result in social isolation, reduced mobility, and loss of social roles, all of which can contribute to feelings of loneliness, which generates a vicious circle.

In this context, our survey explores the significance of low level of loneliness as a protective factor for chronic illnesses among the elderly in correlation analysis and univariate analysis based on the largest database of older people in China. In other words, social connectedness is a protective factor for all chronic diseases except for diabetes, where it presents as a risk factor. In recent research, loneliness is prevalent among a group of 60 elderly individuals who are affected by various chronic conditions, with heart disease patients exhibiting the highest loneliness scores according to Theeke’s study [21]. Loneliness peaks among those aged 15–75 and is linked to both physical and mental health problems. It raises the likelihood of chronic disease among the elderly (OR = 1.41,95% confidence interval = 1.30–1.54). and generates a higher incidence of reported high cholesterol (OR1.31,95%CI:1.18–1.45). Patterson’s study also correlates loneliness with increased overall mortality, particularly from cardiovascular issues [22].

Our result highlight loneliness as a substantial risk element for hypertension among the elderly. The survey conducted among 1880 elderly Malaysians has revealed that loneliness is a significant risk factor for hypertension. Nearly one-third of the participants reported feeling lonely, and the prevalence of hypertension was 39% (95%CI 36.9–41.3). The logistic regression analysis showed a strong association between loneliness and an increased risk of hypertension, with an OR of 1.31 (95%CI 1.04–1.66) and a significance of *p* ≤ 0.05 [23]. Additionally, individuals with hypertension were found to have moderate levels of loneliness but higher levels of social support and self-efficacy in terms of medication adherence. Both loneliness and social support were identified as important factors influencing medication adherence and self-efficacy [24, 25]. The relationship between loneliness and hypertension was significant in the 60–69 age group but not in those aged 70 and above, possibly suggesting varying coping mechanisms or social support structures. Furthermore, the connection between loneliness and hypertension was particularly strong in females (*P* < 0.001), in contrast to the lack of significant association in males (*P* = 0.131), indicating that social connections may be more crucial for women in managing stress and blood pressure.

Low level of loneliness is verified as a risk factor for diabetes and there is no correlation between loneliness and diabetes in female (*P*= 0.06), which is inconsistent with existing evidence [26, 27]. Diagnosis of diabetes among participants in the SSAPUR questionnaire was determined through a simple binary ‘yes or no’ response rather than an assessment of the severity of the condition. Such a simplified approach to data collection may have introduced bias. Furthermore, the social isolation that often accompanies loneliness could lead to a reduction in unhealthy social eating habits. Upon of this, there is no association between loneliness and diabetes among the elderly in rural and urban areas after stratification, could be considered in light of the disparities in healthcare utilization in China [28]. Urban individuals with better access to services may not exhibit a strong relationship between loneliness and diabetes because they’re receiving interventions that help manage the psychological aspects of their condition. Meanwhile, rural individuals, though possibly more prone to loneliness due to fewer social opportunities, may not show this association because other barriers such as poor healthcare access and lower health literacy levels are the predominant factors influencing their diabetes management.

This survey’s findings highlight loneliness as a significant contributor to stomach diseases among the elderly. Within the research, the rate of stomach disease occurrence was 10.8% in the socially isolated group, contrasting with 5.5% in the socially engaged group. Notably, among socially isolated individuals, the incidence of gastropathy was notably higher for those experiencing either no change or increased depression, compared to the control group (*P* < 0.001). Moreover, within the subset of participants experiencing heightened depression, socially isolated patients had an elevated likelihood of encountering stomach issues. Social isolation was identified as a heightened risk factor for peptic ulcer recurrence, although this relationship might be influenced by the absence or alleviation of depression [12]. The Kaplan-Meier curve illustrated a higher cumulative occurrence of peptic ulcers within the depression group compared to the non-depression group over a 36-month span. Through multivariate analysis, a significant association between depression and the onset of gastropathy was identified (HR = 2.520, 95%CI 1.525–3.356). Moreover, the likelihood of experiencing stomach issues was notably elevated in the absence of social engagement (HR = 2.896, 95%CI 1.817–4.228) [29].

The outcomes emphasize the significance of loneliness as a substantial risk factor for cataract and glaucoma in the elderly. Major health conditions influencing the elderly’s health-related quality of life scores encompass hypertension, heart disease, chronic bronchitis, nervous system disorders, and cancer. Notably, cataract significantly impacts the quality of life [30]. Individuals living alone are more prone to reporting normal or poor health, diminished eyesight, challenges in daily activities, as well as impaired memory and mood. Upon accounting for age, gender, income, and education, a connection was established between loneliness and self-reported instances of glaucoma and cataracts. Adjusting for these variables, glaucoma (OR = 1.50, 95%CI 1.03 ~ 2.19) remained notably elevated among lonely individuals. Among individuals living alone, more prevalent health conditions include arthritis and/or rheumatism, osteoporosis, glaucoma, irreversible and/or untreatable retinal diseases, and cataracts [31].

The results of this survey show that loneliness is a significant risk factor for osteoarthrosis in the elderly. After adjusting for age, sex, income, and education, loneliness was still associated with multiple falls and osteoarticular dysfunction. Loneliness is a significant risk factor for self-reported arthritis and rheumatism. For chronic diseases, the incidence of arthritis and (or) rheumatism in loners was 1.36 times higher than that in people who were not alone (OR = 1.36, 95%CI 1.13–1.63) [31]. A study randomly selected 744 osteoarthritis patients over the age of 65 from 13 primary care clinics in Wuhan, China. The results showed that 26.2% of the patients felt lonely. Loneliness is common in Chinese patients with osteoarthritis. Loneliness has a significant impact on the high prevalence rate of osteoarthritis and many negative health consequences, so daily screening, assessment, and intervention of loneliness are particularly important in the primary care environment [32, 33].

The survey outcomes emphasize that loneliness significantly contributes to the risk of chronic lung disease among the elderly. A substantial 63% of chronic obstructive pulmonary disease (COPD) patients experience moderate to severe loneliness [34]. Similarly, a study involving 30 chronic lung disease patients at a university hospital’s outpatient clinic in Turkey found that nearly 97% reported moderate to severe loneliness when assessed with a loneliness scale [35]. Addressing loneliness becomes crucial for COPD patients as it profoundly influences the progression of the disease [36]. Research indicates that social isolation heightens the risk of winter hospitalization for individuals with COPD [37]. Moreover, there’s an evident and independent link between loneliness and diminished health perception in COPD patients. Addressing loneliness among outpatient COPD individuals could potentially enhance their health perception and decrease healthcare utilization [38]. The advent of COVID-19 and the ensuing social isolation measures have profoundly impacted those with chronic respiratory conditions [39].

The results of this survey show that loneliness is a significant risk factor for asthma in the elderly. In a study, it was found that the association between psychosocial problems and self-reported asthma symptoms showed that among children who reported both loneliness and sleep difficulties, children with less than three friends and children with truancy had higher prevalence rates, while self-reported loneliness had the strongest correlation with sleep problems [39]. Asthma and allergy-stricken young individuals often experience a sense of isolation and distinctiveness from their peers [40]. A sample of 755 elderly individuals from southern New Mexico was collected using random number dialing. Participants provided responses on demographics, social isolation, loneliness, social support, and disease diagnoses, encompassing conditions such as diabetes, hypertension, heart disease, liver disease, arthritis, emphysema, tuberculosis, kidney disease, cancer, asthma, and stroke. The outcomes underline that social variables significantly contribute to predicting disease outcomes among the elderly and various ethnic groups [41]. Both asthma and obesity share common symptoms such as fatigue, pain, depression, and anxiety. These symptoms could potentially result in reduced physical activity, social seclusion, and compromised quality of life. These factors, in turn, might contribute to an escalation in morbidity and mortality over time [42].

Loneliness has been identified as a significant risk factor for individuals with malignancy, impacting their mental health, quality of life, and physical well-being. This is particularly evident in individuals with head and neck cancer and ovarian cancer, where social interactions can be severely affected due to the disease and its treatments [43–45]. Emotional support has been shown to mitigate loneliness and improve mental health in those with advanced gastrointestinal cancer [46]. Moreover, a study has found an association between loneliness and an increased incidence of cancer among middle-aged men, even after adjusting for lifestyle and health factors [47]. In our study, the absence of a significant relationship between loneliness and malignancy in older age groups could suggest better psychological or emotional coping strategies as individuals age or perhaps unappropriated sample size affecting statistical outcomes. There is a significant association of loneliness with malignancy (*P* = 0.002) in males, but not in females (*P* = 0.348), indicating that loneliness in men could impact lifestyle choices or stress responses that affect these conditions. As for female, it is noteworthy that a large cross-sectional study of over 3,000 women from the National Health and Nutrition Examination Survey found no direct association between social isolation and cancer mortality. More research is needed to fully understand the complex interplay between loneliness and malignancy in women [48].

The survey findings underline loneliness as a notable risk factor for reproductive system diseases among the elderly. Within the female demographic, those with a history of preeclampsia (PE) exhibited greater levels of depressive symptoms compared to the non-preeclampsia group. These discrepancies persisted even after accounting for age, body mass index, and education, as well as additional adjustments for factors like partners, unemployment, and physical activity. Notably, differences in anxiety, loneliness, marriage quality, optimism, or type D personality were not statistically significant. The increase in blood pressure measured four years prior among individuals in the sports group did not elucidate these differences. Moreover, within the PE group, those who experienced stillbirth or early neonatal death during the index pregnancy reported higher levels of depressive symptoms, anxiety, fatigue, and loneliness. However, these psychosocial factors explained only a fraction of these differences [49].

Our study contains inherent limitations that should be considered when interpreting its findings. Primarily, the assessment of patients’ loneliness and chronic health conditions was based on self-report, which may introduce subjective biases into the data. Future research would benefit from employing widely recognized, multi-faceted, and professionally-developed scales to measure loneliness more accurately. Additionally, the determination of chronic disease status would be more reliable if it were corroborated with actual medical records from healthcare institutions, given the varying degrees and classifications of chronic illness. Overall, further research is required to refine the measurement of loneliness and to establish a more objective evaluation of chronic disease conditions, which will enhance the understanding of such complex association.

Last, it is essential to emphasize the potential implications of the findings derived from this large-scale urban and rural elderly sample survey from China, in conjunction with similar studies conducted internationally. Loneliness has evidently emerged as an independent risk factor for chronic diseases that warrants intervention. Given that our research is cross-sectional in nature, future directions should pivot towards primary prevention, exploring the impact of early identification and subsequent medical and social interventions on the elderly who experience loneliness, on the outcomes related to chronic diseases. Moreover, there is an opportunity to integrate loneliness into clinical diagnostic protocols by considering it as a part of the chronic disease grade assessment to explore differences in chronic disease prognoses. All endeavor contributes to the advancement of quality of life in aging societies.

## Conclusion

To conclude, loneliness emerges as a crucial risk element contributing to the onset and progression of chronic illnesses among the elderly in China. Its detrimental impact is evident across conditions such as hypertension, stomach disease, cataract, glaucoma, osteoarthrosis, chronic lung disease, asthma, malignant tumors, and reproductive system diseases. Consequently, regular screening, assessment, and intervention for loneliness hold exceptional significance within the primary healthcare setting.

### Electronic supplementary material

Below is the link to the electronic supplementary material.

Supplementary Material 1

Supplementary Material 2

Supplementary Material 3

Supplementary Material 4

## Data Availability

The datasets used and/or analyzed during the current study are available from the corresponding author on reasonable request.
