# Supplementary material for: The silent epidemic: exploring the link between loneliness and chronic diseases in China’s elderly
Source: BMC Geriatr. 2024 Aug 26;24:710. doi: 10.1186/s12877-024-05163-2 (PMC11346041; doi:10.1186/s12877-024-05163-2)
Supplement: Supplementary file 1 — Supplementary Material 1 [file 12877_2024_5163_MOESM1_ESM.pdf]

11.11

北京医院伦理委员会

伦理审查意见

伦理审查意见号<br>Approval Letter No.	2021BJYYEC-294-01		
项目全称<br>Study Title	心理及其相关因素对老年健康的影响及机制——心理及其相关因素对老年人慢性疾病的影响		
项目来源<br>Study Source	中华人民共和国科学技术部		
研究单位<br>Study Institution	北京医院		
主要研究者<br>Principle Investigator	刘德平		
审查日期<br>Review date	2021-11-15	审查地点<br>Address	伦理委员会办公室
审查类别<br>Review type	初始审查申请	审查方式<br>The Way of Review	快速审查
主　审<br>Review Member	毛永辉 赵亚光		
审查决定<br>Result	同意		
同意文件<br>Approval Documents	1.初始审查申请<br>2.研究方案--中文（注明版本号和版本日期）（版本号：1.0版，版本日期：2021-11-01）<br>3.免除知情同意说明<br>4.主要研究者简历<br>5.主要研究者资质<br>6.研究团队列表及分工<br>7.问卷1<br>8.科研项目批文_任务书		
审查意见<br>Review Opinions	经本伦理委员会审查，同意按照上述同意文件开展本项研究。		
跟踪审查频率<br>tracking review	6 个月		

frequency	
伦理审查意见有效期<br>Expiration Date	2022-11-15
联系人与联系电话<br>Contact and contact number	张弼　　010-85138105
主任委员/副主任委员/会议主持人签字<br>Director/Vice Director Signature/Stamp	
北京医院伦理委员会（盖章）<br>IRB	北京医院 伦理委员会 11000[illegible]
日期<br>Date	2021-11-15　2021-11-15
声明及注意事项<br>Statement	1、北京医院伦理委员会人员组成、职责分工、操作规程均遵循国家相关法律法规、指导原则和 GCP 的规定，且伦理委员会是独立的，成员均签署保密协议和利益冲突声明。<br>2、请按照 GCP 原则和伦理委员会同意的文件开展研究，保护受试者的健康与权益。<br>3、凡涉及人类遗传资源管理或者国家规定必须经有关部门审批的项目，均需在执行前向有关部门申报并获得批准；涉及人的生物医学研究在实施前，需要在医学研究登记备案信息系统进行备案。<br>4、对已同意的研究方案、知情同意书（如有）、招募材料（如有）等的任何修改以及主要研究者的变更，请提交修正案审查申请。<br>5、发生需要提交的安全性事件时，请及时提交。<br>6、无论研究开始与否，请按照伦理委员会规定的跟踪审查频率，在截止日期前 1 个月提交研究进展报告。<br>7、暂停或提前终止研究，请及时提交暂停/终止研究报告。<br>8、完成临床研究，请提交结题报告。<br>9、研究过程中，当出现任何可能显著影响研究进行或增加受试者风险的情况，请立即向伦理委员会提交书面报告。

伦理委员会地址：北京市东城区东单大华路 1 号，邮编：100730
