# Supplementary material for: The silent epidemic: exploring the link between loneliness and chronic diseases in China’s elderly
Source: BMC Geriatr. 2024 Aug 26;24:710. doi: 10.1186/s12877-024-05163-2 (PMC11346041; doi:10.1186/s12877-024-05163-2)
Supplement: Supplementary file 2 — Supplementary Material 2 [file 12877_2024_5163_MOESM2_ESM.docx]

Printing serial number:

Questionnaire No. :

| Article 7 of the Statistics Law of the People's Republic of China stipulates: State organs, enterprises, institutions and other organizations and individual businesses and individuals under statistical investigation, must be in accordance with this law and relevant regulations of the state, true, accurate, complete and timely provide the necessary statistical investigation data, shall not provide false or incomplete statistics, statistical data shall not be leaders and organizations  Article 25 of the Statistical Law of the People's Republic of China stipulates: No unit or individual may provide or divulge the data obtained during statistical investigations that can identify or deduce the identity of individual statistical objects and may not be used for purposes other than statistics |
| --- |

Table no. : CRCA2015-1

Formulating authority: Ministry of Civil Affairs

Approving authority: National Bureau of Statistics

Approval Number: National Control [2014] No. 87

Valid until: September 2015

The fourth sampling survey on the living conditions of the elderly in urban and rural China

Personal questionnaire (long form)

*********************************************************************

I. Place of visit:

Provinces (autonomous regions, municipalities directly under the Central Government) _____________________________________________________________________

Prefecture level ________________________________________________________

County (city, district) ___________________________________________________

Township/Sub-district ___________________________________________________

Village (neighborhood) committee _________________________________________

Home address _________________________________________________________

II. Visit Records:

| Visit date | | The start time | The end of time |
| --- | --- | --- | --- |
| Month | Day |  |  |
|  |  |  |  |
|  |  |  |  |
|  |  |  |  |

Investigator's signature: _________________ Telephone: _________ Date: __ Month ___Day

Signature of township supervisor: _________ Telephone: _________ Date: __ Month ___ Day

Signature of county supervisor: ___________ Telephone: _________ Date: __ Month ___Day

Signature of input personnel: _____________ Telephone: _________ Date: __ Month ___Day

【 Search the guide words 】

Dear __________ (name of the old man interviewed):

I am a staff member of the fourth survey of the living conditions of the elderly, which is a major event in the life of the elderly nationwide and has been approved by the National Working Committee on Aging and the National Bureau of Statistics. We hope that through this survey, we will have a comprehensive understanding of the various situations, difficulties and problems in the life of the elderly nationwide, and provide a basis for the Party and the government to formulate policies, with the aim of improving the quality and level of life of the elderly nationwide. You have the honor to represent the nation's senior citizens. We will strictly abide by the relevant provisions of the Statistics Law and do a good job of confidentiality work. We hope you can tell us the actual situation of your life. Thank you very much for your cooperation!

[Fill in the form]

| 1. The questionnaire shall be filled out by the investigators at home. All the data must be fair, objective and accurate. 2. There is no right or wrong answer to this questionnaire, but it must accurately reflect the real situation of the interviewees. 3. There are two types of questions in this questionnaire: one is multiple choice questions, and the other is blank filling questions. 4. Single choice: Please tick one of the answers.   5. Multiple choice: Please tick the box in □ in front of the corresponding choice.  6. Fill in the blanks: Please fill in the answers (words or figures) that reflect the true situation of the interviewee.  7. If you need to specify in detail when selecting "Others", please give corresponding explanation.  8. If some questions are not applicable, the elderly cannot answer or refuse to answer, please mark "×" in the number of questions and mark the reason beside the number of questions. |
| --- |

1. Basic situation

A1 Gender of the elderly interviewed: [Investigator Observation and Fill in]: 1 male 0 female

A2 Date of birth: [Investigator to fill in according to ID card] _____ Year _____ Month

A3 What kind of domicile do you have? [Investigators fill in according to the household registration book]

1. Agricultural 2. Non-agricultural 3. Unified household registration

A4 What nationality do you belong to?[Investigator to fill in according to ID card]

1 The Han nationality 2 The Zhuang nationality 3 The Hui nationality

4 The Man nationality 5 The Uygur nationality 6 The Miao nationality 7 The Yi nationality 8 The Tujia nationality 9 The Tibetan nationality 10 The Mongolian nationality 11 other nationalities __________________ (please write the full name of the nationality)

A5 Your education level:

1 never attended school (including literacy classes) 2 Primary school culture (including private schools) 3 Junior high school culture 4 High school/technical secondary school/vocational high school culture 5 University college 6 Bachelor degree or above

A6 Your professional and technical title: 1 No 2 Technician Level 3 Junior Title 4 Intermediate Title 5 Senior Title

A7 Your political status: 1 general public 2 members of the Communist Party of China

3 democratic parties 4 personages without party affiliation

A8 Your current marital status:

1. Has a spouse whose age is ________?
2. 2 widowed, widowed ________ years ago?

3 Divorce, Divorce ________ years ago?

4 Never married

B Family situation

[Investigator: Next, we want to know about your family.]

B1 Status of your current children: [Investigator: including foster/stepchildren, excluding daughter-in-law/son-in-law]

B1.1 Son _____ (The number of person);

B1.2 daughter _____ (The number of person).

[If the respondent has no children, please ask the questions of B2 directly]

B1.3 Do any of your children have difficulties in life?

1 Yes 0 No [If the child of the respondents do not have living difficulties, please ask the questions of B1.5 directly]]

B1.4 Do you support your child financially on a long-term basis?

1 Yes 0 No

B1.5 Do your children take turns taking care of you?

1 Yes 0 No

B1.6 Do you think your children are filial?

1 filial piety 2 general 3 not filial piety

B2 Who are in your family (living with you) now?(Multiple choice)

[Investigator: select according to the relationship with the elderly interviewed, and indicate the number of people]

□ Living alone □ Spouse □ The Parents -in-law/Parents _____ (The number of person)□ Son _____ (The number of person)□ daughter-in-law _____ (The number of person) □ daughter _____(The number of person)□ son-in-law_____ (The number of person)□ (Great) grandchildren _____(The number of person)□ nanny_____ (The number of person) □ other people_____(The number of person)

B3 Do you have children residing outside this province?

1 Yes 0 No [If the child of the respondent does not reside outside the province, please ask the questions of B4 directly]

B3.1 How many children do you have living outside this province? __________ (The number of person)

B3.2 How many times do your children, who live outside the province, visit you each year?

1 less than once 2 once 3 two or three times a 4 more than four times

B4 Are you willing to live with your children for a long time?

1 Yes 2 No 3 Depends

B5 Do you now help your children with the following things? (Multiple choice) [Investigator: Question item by item]

□ Take care of the home □ Do housework *(rural)

□ Do farm work □ Take care of grandchildren

□ Others (please specify) _________□ None have been done

B6 How many relatives/friends do you keep up with regularly?

B7 What major events have happened to your family since this year?(Multiple choice) [Investigator: Question item by item]

□ Children of unemployment □ Children of divorce

□ Disputes/Lawsuits □ Relatives of a serious illness

□ Loved ones death □ Others (please specify) _____

□ None

B8 What kind of transportation do you usually use?(Multiple choice) [Investigator: Question item by item]

□ By bike □ By motorcycle/electric bike

□ By bus □ By subway

□ By taxi □ By car

□ Others (please specify) __________ □ None

B9 Who has the final say on major expenses in your family?

1. self 2 spouse 3 children 4 negotiate together

C. Health and medical conditions

[Investigator: Next, we'd like to know about your medical condition]

C1 Do you smoke?

1. Never [If the respondent does not smoke, please ask the questions of C2 directly]

2. I used to smoke, but now I have given up smoking

3 Often

4 Occasionally

C1.1 How many years have you smoked? _____

C2 Do you drink alcohol?

1. not drinking or occasionally 2 .1-2 times a week

3. at least 3 times a week 4 .often drunk

C3 How do you usually sleep?

1. very good 2 relatively good 3 average

4 relatively poor 5 very poor

C4 Can you see clearly (including glasses)?

1 very clear 2 relatively clear 3 generally 4 not so clear 5 almost/completely unclear

C5 Can you hear clearly (including the hearing aid)?

1 Difficult to hear clearly 2 Need someone to raise their voice 3 Can hear clearly

C6 Do your teeth affect your eating?1 makes a difference and 0 doesn't make a difference

C7 Do you often feel pain?

1 Yes 0 No [If the respondent doesn't often feel pain, please ask the questions of C8 directly]

C7.1 If you often feel pain, how bad is the pain?

1 not serious 2 moderate 3 serious

C8 How many times a week do you work out?

1 never 2 less than once 3 one or two times

4 three to five times 5 six times or more

C9 Do you take any health supplements?

1 Never 2 Occasionally 3 Often

C10 Did you have a medical examination in 2014?

1 Yes 0 No

C11 Do you suffer from any of the following chronic diseases? (Multiple choice) [Investigator: Question item by item]

□ Cataract/glaucoma □ Hypertension □ Diabetes

□ Cardiovascular and cerebrovascular diseases (coronary heart disease/angina pectoris/stroke, etc.) □ Stomach diseases

□ osteoarthropathy (osteoporosis/arthritis/rheumatism/disc disease, etc.)

□ Chronic lung diseases (COPD/tracheitis/emphysema, etc.) □ Asthma

□ Malignant tumors □ Reproductive system diseases

□ Other chronic diseases (please specify) __________ □ None

C12 Were you ill in the two weeks prior to the survey?

1 Yes 0 No [If the respondent had never been ill, please ask the questions of C13 directly]

C12.1 What kind of illness do you have this time?

1 .It's new in two weeks 2 .The acute disease began two weeks ago and continued for two weeks

3. The onset of chronic disease began two weeks ago and continued within two weeks

C12.2 How did you deal with it after you fell ill?

1. To cure 2. Not dealing with it [If the respondent fell ill and was not treated, please ask the questions of C12.4 directly] 3. self-medication [If the respondent fell ill, self-treatment was adopted, please ask the questions of C12.5 directly]

C12.3 How many times have you been to the hospital or clinic in the last two weeks?_______ times [Jump ask C13]

C12.4 What's the main reason you didn't dispose of it?

(Multiple choice) [Investigator: Question item by item]

□ light self-induced illness □ economic difficulties □ don't have the time □ mobility inconvenience□ Unaccompanied □ Hospital too far away □ Trouble for medical treatment □ Other reasons (please specify) __________[Answer this question and skip to C13]

C12.5 Which of the following self-therapy measures have you taken?

(Multiple choice) [Investigator: Question item by item]

□ Buy your own medicine □ Use traditional methods of treatment □ Use health care and rehabilitation equipment□ Others (please specify) __________

C13 Where do you usually see your doctor?

[Investigator: I may have visited multiple medical and health institutions, and the medical and health institutions I filled in the most]

1 private clinic 2 clinics/stations 3 community health service centers 4 township/sub-district health centers5 county/city/district hospitals 6 city/prefecture hospitals 7 provincial hospitals 8 others (please specify) __________

C13.1 How far is the health care facility you visit most from your home?

1. less than 1 km

2. 1to 2 km

3 .3 to 5 km

4 .5 km and above

C14 Have you encountered any of the following problems when visiting a hospital or clinic?(Multiple choice) [Investigator: Question item by item]

□ The queue time is too long □ The formalities are too tedious □ The barrier-free facilities are not sound □ Can not be hospitalized in time□ The service attitude is not good □ The charge is too high □ Others (please specify) __________

C15 How many times were you hospitalized in 2014? ______ times

C16 How much did your hospital/hospital expenses total in 2014?______RMB..

C16.1 Among them, at one's own expense (can not be reimbursed) spent how much money? _____RMB...

C16.2 How much does your child or other person pay for you at one's own expense? ________RMB

C17 How much did you spend on out-of-pocket medications at the drugstore in 2014? _____RMB...

C18 Which of the following medical benefits do you enjoy?

(Multiple choice) [Investigator: Question item by item]

□ Basic medical insurance for urban workers □ Basic medical insurance for urban residents □ New rural cooperative medical insurance□ Basic medical insurance for urban and rural residents (urban residents' basic medical insurance is integrated with the new rural cooperative medical insurance)□ Serious disease insurance for urban and rural residents □ large medical subsidies for employees□ Free medical □ Others (please specify) __________ □ None

C18.1 Do you think it is convenient to reimburse medical expenses?

1 very convenient 2 more convenient 3 general

4 more inconvenient 5 very inconvenient

C19 Do you have commercial health insurance?

1 Yes 0 No

C20 How do you feel about your health?

1. very good 2 relatively good 3 average
2. 4 relatively poor 5 very poor

D Status of care services

[Investigator: Next, we'd like to know about your care services.]

D1 Which of the following daily activities do you do?

| Daily activities | Can do it | Some difficulties | Can't do it |
| --- | --- | --- | --- |
| 1 Have a meal | 1 | 2 | 3 |
| 2 Dress | 1 | 2 | 3 |
| 3 Go to the toilet | 1 | 2 | 3 |
| 4 Fluctuation bed | 1 | 2 | 3 |
| 5. Walk indoors | 1 | 2 | 3 |
| 1. Have a bath | 1 | 2 | 3 |
| 7 Cooking | 1 | 2 | 3 |
| 8 Do the laundry | 1 | 2 | 3 |
| 9 Sweep the floor | 1 | 2 | 3 |
| 10 Daily shopping | 1 | 2 | 3 |
| 11 Going up and down the stairs | 1 | 2 | 3 |
| 12 Taking a bus | 1 | 2 | 3 |
| 13 Lift a weight of 5 kg | 1 | 2 | 3 |
| 14 Call up | 1 | 2 | 3 |
| 15.Manage personal finances | 1 | 2 | 3 |

D2 Many older people have incontinence, do you have it?

(Multiple choice) [Investigator: Question item by item]

□ fecal incontinence □ urine incontinence □ none

D3 Are you using any of the following accessories now?

(Multiple choice) [Investigator: Question item by item]

□ Reading glasses □ hearing AIDS □ dentures □ crutches □ wheelchair □ blood pressure monitor□ Glucose meters □ adult diapers/nursing MATS □ massage appliances □ smart wearables□ nursing bed □ others (please specify) __________ □ none

D4 Do you need someone to take care of your daily life now?

1 need 0 don't need [jump to ask the question of D5]

D4.1 Do you have any caregivers?

1Yes 0 No [jump to ask the question of D5]

D4.2 Who is your primary caregiver?

1 spouse 2 son 3 daughter-in-law 4 daughter 5 son-in-law

6 grandchildren 7 other relatives 8 friends/neighbours 9 volunteers

10 domestic service personnel (nannies, hourly workers, etc.)

11 personnel in medical and nursing institutions

12 nursing home staff 13 community workers 14 others (please specify)__________

D4.3 How old is she/he (primary caregiver)? Age of the___...

D5 Are there any other elderly people in your family who need to be cared for?

1Yes 0 No[jump to ask the question of D6]

D5.1 Who is taking care of him/her now?

1 the elderly interviewed 2 others (please specify)__________

D6 Where would you most like to receive care if needed?

1 at home[jump to ask the question of D7] 2 in the community during the day home at night[jump to ask the question of D7] 3 in a nursing home 4 as the case may be

D6.1 If you live in an assisted-living facility, what is the maximum monthly expenses you (and your family) can afford?

1. 1000 yuan below 2 .1000-1999 yuan 3. 2000-2999 yuan

4. 3000-3999 yuan 5. 4000-4999 yuan 6. 5000 yuan and above

D7 Your need, knowledge and utilization of the following community service programs for the aged: [Investigator: item by item inquiry]

| Service project | whether need | whether there is | Whether to use |
| --- | --- | --- | --- |
| 1.Meal assistance service | 1 Yes 0 No | 1 Yes 2 No  3 Don’t know | 1 Yes 0 No |
| 2. Bath services | 1 Yes 0 No | 1 Yes 2 No  3 Don’t know | 1 Yes 0 No |
| 3. Do housework | 1 Yes 0 No | 1 Yes 2 No  3 Don’t know | 1 Yes 0 No |
| 4. Visit a doctor | 1 Yes 0 No | 1 Yes 2 No  3 Don’t know | 1 Yes 0 No |
| 5 Day Care | 1 Yes 0 No | 1 Yes 2 No  3 Don’t know | 1 Yes 0 No |
| 6 Rehabilitation nursing | 1 Yes 0 No | 1 Yes 2 No  3 Don’t know | 1 Yes 0 No |
| 7. Auxiliary appliances and supplies for the elderly | 1 Yes 0 No | 1 Yes 2 No  3 Don’t know | 1 Yes 0 No |
| 8.Health education services | 1 Yes 0 No | 1 Yes 2 No  3 Don’t know | 1 Yes 0 No |
| 9.Psychological consultation/chat to relieve boredom | 1 Yes 0 No | 1 Yes 2 No  3 Don’t know | 1 Yes 0 No |

E. Economic status

[Investigator: Next, we want to know the basic financial situation of your personal and family.]

E1 Have you gone through the retirement formalities now?

1 Yes 2 No [Jump to ask the question of E2] 3 Not applicable (Never had a formal job) [Jump to ask the question of E2]

E1.1 How old were you when you retired? Age of the_____...

E1.2 Are you retiring early? 1 Yes 2 No

E1.3 What is the nature of your work unit before retirement?

1 Party and government organs 2 public institutions 3 state-owned enterprises 4 collective enterprises 5 private enterprises 6 three types of foreign-funded enterprises 7 troops 8 rural collectives 9 others (please specify) __________

E2 Are you still engaged in gainful employment (including working, doing business, etc.)?

1 Yes 0 No[Jump to ask the question of E2.3]

E2.1 How did you get the job?

1 personal relationship 2 company reemployment 3 market recruitment 4 government help 5 own business 6 others (please specify) __________

E2.2 What was your income from the above work last month? ________RMB

E2.3 Are you willing to engage in gainful employment (including working, doing business, etc.)? 1 Yes 0 No

E3 * (Rural) Are you engaged in agriculture, forestry, animal husbandry, sideline fishing and other economic activities?

1 Yes 0 No. [Jump to ask the question of E4]

E3.1What was your net income from the above economic activities in 2014? ________RMB...

E4 Have you and your wife saved any money for retirement?

1 Yes 0 No. [Jump to ask the question of E4]

E4.1 How much money is there? _____RMB...

E5 Do you have the following monthly income now?

[Investigator: ask item by item, if there is, fill in the specific amount, if there is no, fill in 0]

E5.1 annuity（pension）____yuan E5.2 Survivors' benefits ____ yuan E5.3 occupational annuity / enterprise annuity ___________ yuan E5.4 Commercial pension ________ yuan

E5.5 old age allowance ________ yuan E5.6 Pension service subsidy__________ yuan

E5.7 Care subsidies________ yuan E5.8 Minimum living allowance________ yuan

E5.9 Five insurance subsidies / No rescue subventions _______ yuan

E5.10 Reward (special) subsidy for family planning families ________ yuan

E5.11 Other Social Security Income (please specify)__________，__________ yuan

E6 Do you and your wife have the following income in 2014? [Investigator: ask item by item, if there is, fill in the specific amount, if there is no, fill in 0]

E6.1 The income of rent is ________ yuan

E6.2 The income of interest is ________ yuan

E6.3 The income of * (rural) land leasing/contracting is ______yuan

E6.4 The income of original unit welfare/collective subsidy/bonus is _________ yuan

E6.5 The money given by children (grandchildren) (including in kind) ________ yuan

E6.6 The money given by other relatives ________ yuan (including the kind)

E7 Which of the following investment and financial activities are you engaged in now?

(Multiple choice) [Investigator: Question item by item]

□ Treasury bonds/bonds □ stocks □ funds □ foreign exchange □ precious metals

□ Other financial products □ Other (please specify) __________ □ None[Jump to ask the question of E8]

E7.1 If there are the above financial assets, the total present value is ________ million yuan

E8 Do you own (or your spouse's) home?

1 Yes 0 No. [Jump to ask the question of E9]

E8.1 How many apartments are there?___...

E8.2 What is the approximate value of these houses now?

________ten thousand yuan...

E8.3 Would you be willing to sell/rent/mortgage your house for a pension?

1 yes, 2 no, 3 depends

E9 What kind of housing do you live in now?

1. Owned property right 2. Children's property

3. Grandchildren's property 4 Renting public house

5 Rent private house 6 Borrow 7 Others (please specify) __________

E10 Your average monthly daily living expenses:

[Investigator: ask item by item, if there is, fill in the specific amount, if there is no, fill in 0]

E10.1 The expenditure of personal articles (including tobacco and alcohol, cosmetics, toiletries, etc.) is ________ yuan

E10.2 The expenditure of transportation is ________ yuan

E10.3 The expenditure of communication is ________ yuan

E10.4 The expenditure of Hire nanny/hour worker/caregiver is_yuan

E10.5 The expenditure of Health care (beauty salons, health care products, massage, etc.) is ________ yuan

E10.6 The expenditure of recreational, sports and entertainment (watching movies, books and newspapers, etc.) is ________ yuan

E11 The following information about your personal expenses in 2014: [Investigator: ask item by item, fill in the specific amount if there is any, or fill in 0 if there is no]

E11.1 The expenditure for clothes, shoes and hats is ________ yuan

E11.2 The expenditure for tourism is ________ yuan

E11.3 Give ___yuan to children/grandchildren

E11.4 The expenditure for buy assistive equipment (denture, wheelchair, hearing aid, etc.) is ________ yuan

E12The following expenditure information of you and your wife（husband）in 2014:

[Investigator: ask item by item, if there is any, fill in the specific amount; if there is no, fill in 0]

E12.1 The house rent is________________yuan

E12.2 The heating fee is ____________ yuan

E12.3 The Property management fee is ______________ yuan

E12.4 The cost for the house/decoration is________ million yuan

E12.5 The cost of furniture and appliances is ________ yuan

E12.6 The cost of buy vehicles is __________ yuan

E12.7 The cost of expensive jewelry is ____________ yuan

E13 The average monthly expenditure on food (board) in your family is _____________ yuan

E14 The total expenditure of your family in 2014 is ____million yuan

E15 In 2014, the total income of your family is _______million yuan

E16 How much debt does your family have right now?_____ yuan

E17 Do you think your grandchildren/children are "mooching off" their parents money?

1 Yes 0 No.

E18 Which of the following would you consider your financial situation to be?

1 very well off 2 relatively well off 3 basically enough 4 relatively difficult 5 very difficult

F Conditions of livable environment

[Investigator: Next, we want to know about your livable environment]

F1 When was the house you are living in built?

1. Before liberation 2.1950s-1960s 3. 1970s-1980s

4. In the 90 s 5. after 2000s

F2 What is the total floor area of your house now?

__________ the square meters

F3 Do you have a separate room (with your wife)? 1 Yes 0 No

F4 Do you have the following living facilities in your present apartment?(Multiple choice) [Investigator: Question item by item]

□ tap water □ gas/natural gas/biogas □ heating

□ Indoor toilets □ Bath/shower facilities □ None

F5 Do you have any of the following electronics and household appliances in your current house?(Multiple choice) [Investigator: Question item by item]

□ fixed-line phone □ elderly mobile phone □ smart phone □ ordinary mobile phone □ computer □ TV□ washing machine □ air conditioner □ refrigerator □ air purifier □ water purification equipment □ None

F6 Have you fallen down this year?

1 Yes 0 No[Jump to ask the question of F7]

F6.1 Where was the last time you fell?

1 bedroom 2 bathroom 3 living room 4 kitchen

5 balcony 6 threshold 7 stairs/steps

8 courtyards 9 roads 10 vehicles 11 shopping places 12 fitness places

13 parks 14 workplaces 15 other locations (please specify) _____

F6.2 What are the consequences of your fall?

1 no injury 2 minor injuries, no medical attention 3 serious injuries, requiring medical attention 4 serious injuries, long term bed

F7 Which of the following conditions exists in your current residence?(Multiple choice) [Investigator: Question item by item]

□ dim light □ stumbling threshold or uneven floor □ no handrails □ slippery floor

□ Door doesn't work properly □ Toilet/bathroom doesn't work well □ No calling/alarm facilities

□ There is noise □ Others (please specify) __________ □ Everything is fine, no problem

F8 Are you satisfied with your present living conditions?

1 Satisfied 2 General 3 Not satisfied

F9 How many years have you lived in this community (village/house)

F10 What kind of relationship do you have with your neighbors?

1 Don't know 2 Just say hello

3 Communicate and keep in touch 4 Help each other when necessary

F11 Which of the following are you satisfied with in your community (village/residence)?(Multiple choice)

□ Signage □ Road/street lighting □ Traffic conditions □ Household facilities

□ Fitness place □ Public toilet □ Green environment □ Public security environment

□ The atmosphere of respecting the elderly □ are not satisfactory

G Social participation

[Investigator: Next, we want to know about your social participation]

G1 Do you often participate in the following public benefit activities?(Multiple choice)

□ Safeguard community public order □ Help mediate neighborhood disputes □ Safeguard community health environment□ Help neighbors □ Care about educating the next generation (not including educating your grandchildren)

□ Participating in cultural and scientific promotion activities □ None

G2 Which of the following organizations or groups do you belong to?

(Multiple choice) [Investigator: Question item by item]

□ Community Security Group □ People's Mediation Committee

□ Social welfare organizations (volunteer/charity, etc.) □ Cultural, sports and entertainment organizations (painting/singing/dancing, etc.)□ Folk/folk cultural organizations □ Professional and technical groups or organizations□Cooperative organizations for the elderly (voluntary pension groups/economic organizations for the elderly)

□ Other organizations (please specify) __________ □ None

G3 Have you attended any of the following clan/clan events?

(Multiple choice) [Investigator: Question item by item]

□ Repair family tree/genealogy □ Take part in ancestor worship activities □ Take part in charity/public welfare activities organized by the family/clan□ Help to mediate intra-clan or inter-clan disputes □ Other activities (please specify) __________ □None

G4 Are you a member of the senior society?

1 Yes 0 No. [Jump to ask the question of G4.3].

G4.1 Are you satisfied with the activities organized by the senior society?

1 Very Satisfied 2 Be fairly satisfied 3 Generally Satisfied

4 Less satisfied 5 Very Unsatisfied

G4.2 What activities would you like the Senior Society to carry out?

(Multiple choice) [Investigator: Question item by item]

□ study/entertainment activities □ help activities for the elderly in difficulties □ family activities for the old and the young□ protection of the rights and interests of the elderly □ voluntary public welfare activities □ profit-making project activities□ participate in community public affairs □ others (please specify) __________ □ no suggestion [Jump to ask the question of G5]

G4.3 What is the main reason you don't join the senior society?

(Multiple choice) [Investigator: Question item by item]

□ Not established □ Not interested □ No time □ Not allowed by health

□ Family members do not support □ Others (please specify) ___

G5 Are you willing to help the elderly in your community who are in need? 1 Yes 0 No

G6 Did you take part in the last community election? 1 Yes 0 No

G7 Are you concerned about community affairs being open?

1 cares about 2 doesn't care about 3 It doesn't matter

G8 Have you ever been consulted on important projects in our community? 1 Yes 0 No

G9 Have you made suggestions to the community? 1 Yes 0 No

G10 Do you care about state affairs? 1 Yes 0 No

H Rights Protection Status

[Investigator: Next, we want to know about your rights protection status]

H1 Do you know the <Law on the Protection of the Rights and Interests of the Elderly>?

1 Yes 0 No

H2 Have you got a privilege card for senior citizens? 1 Yes 0 No

H3 Have you ever enjoyed any of the following preferential treatment for the elderly?(Multiple choice) [Investigator: Question item by item]

□ Free physical examination □ Remission of the registration fee for general outpatient service□Remission of public transport tickets □ Remission of park tickets□ Ticket reduction or exemption for tourist attractions □ Ticket reduction or exemption for public cultural places such as museums and public libraries□None

H4 Has your family treated you in any of the following ways this year?

(Multiple choice) [Investigator: Question item by item]

□ Not provided when you ask for basic living expenses□The accommodation provided to you is in poor condition□You are not well fed/poorly fed□No medical treatment for you □ Not taking care of you when you need it□ encroaching on your property □ hasn't come to visit/greet/talk to you for a long time□ often beat and scold you * (only for widowed or divorced elderly people) □ prevent your remarriage□ Other actions (please specify) ___□ None[Jump to ask the question of H5]

H4.1 If one of the above situations exists, what measures have you taken to resolve it? (Multiple choice)

□ I've been wronged/swallowed my pride □ I've sought mediation from relatives/clans□Go to the neighborhood committee for help. □Go to the old people's association for help□ find family unit mediation □ lawsuit/find the judicial authority to solve

□ Report to the media □ Others (please specify) __________

H5 Which of the following situations have you encountered so far this year? (Multiple choice) [Investigator: Question item by item]

□ be cheated cheated □ be robbed □ be stolen

□ be beaten/threatened □ others (please specify) _______□ none

H6 Have you received any legal aid this year? 1 Yes 0 No

H7 Do you think your legal rights and interests are properly protected? 1 Yes 0 No

I. Spiritual and cultural life

[Investigator: Next, we want to know about your spiritual and cultural life]

I1 Do you often take part in the following activities?

(Multiple choice) [Investigator: Question item by item]

□Watch TV/listen to the radio □ read books/read newspapers□ go to the cinema/to the theatre □go for a walk/jog, etc□ Tai Chi/Health exercises etc.□Dancing (Square dance/Yangko dance)□Play gateball/table tennis/badminton etc.□Play mahjong/cards/chess etc

□ grow flowers and grass. □ keep pets

□ Fishing/Calligraphy/Photography/Collection □ Others (please specify) __________□None

I2 Do you often surf the Internet?

1 Yes 0 No [Jump to ask the question of I3]

I2.1 If you often surf the Internet, do you do the following?

(Multiple choice) [Investigator: Question item by item]

□ watching news □ watching movies and TV series □ chatting □ shopping□ Play games □ Fry stocks □ Others (please specify) __________

I3 Have you attended a university/school for the elderly (including distance education for the elderly)? 1 Yes 0 No

I4 Are there any of the following activities near your home?Do you often go to activities?

[Investigator: item by item inquiry. If the interviewed old people answered "no" or "don't know", they would not ask whether they often go there.]

| Activities | Have any place | How often do you go |
| --- | --- | --- |
| 1.Square | 1 Yes 2 No  3 Don’t know | 1 Never 2 Occasionally  3 Usually |
| 2.Park | 1 Yes 2 No  3 Don’t know | 1 Never 2 Occasionally  3 Usually |
| 3.Fitness centers | 1 Yes 2 No  3 Don’t know | 1 Never 2 Occasionally  3 Usually |
| 4.Senior citizens activity center | 1 Yes 2 No  3 Don’t know | 1 Never 2 Occasionally  3 Usually |
| 5.Library | 1 Yes 2 No  3 Don’t know | 1 Never 2 Occasionally  3 Usually |

I5 通常您每天用于下列活动的时间分布情况是怎样的？

| Activity type | Activity time |
| --- | --- |
| 1.Gainful work/Labour/business activity | ___________________hours |
| 2.Housework | ___________________hours |
| 3.Watch television | ___________________hours |
| 4.Reading books and newspapers | ___________________hours |
| 5.Other leisure activities | ___________________hours |
| 6.Midday rest | ___________________hours |

I6 Do you have any plans to travel in the coming year?

1 Yes 2 No 3 Not sure

I7 What religion do you follow now?

1 Belief in no religion 2 Buddhism 3 Islam

4 Christianity 5 Catholicism 6 Taoism

7 Other religions (please specify) __________

I8 Have you ever seen the following situations in your daily life?

(Multiple choice) [Investigator: Question item by item]

□Sudden strangeness to the faces of relatives and friends □ often cannot remember the names of relatives and friends□Unable to find my own house when I go out □ I often forget to bring my keys□I often forget that porridge or water is boiling on the stove□None

I9 Do you feel lonely? 1 Often 2 Sometimes 3 Never

I10 In the past week, have you had the following feelings?

(Multiple choice) [Investigator: Question item by item]

□You feel happy most of the time. □You feel fidgety and restless all day

□You feel low all the time. □You think it's good to be alive now

I11 What is your mental age? Age of the___

I12 For various reasons, What do you think of a few old people have committed suicide?

1.Cherish life 2. let nature take its course 3 have the right to give up life

I13 Do you agree with the following statement?

(Multiple choice) [Investigator: Question item by item]

□The elderly should give full play to their spare energy and participate in social development□Elderly people should enjoy life and be supported by their families and society□Old people are a burden on their families□Old people are a burden on society□The elderly are a valuable asset to the country and society□Elderly people should be self-reliant and do their best not to cause trouble to their children and society

I14 On the whole, do you think you are happy?

1 very happy 2 relatively happy 3 average

4 relatively unhappy 5 very unhappy

***************************************************************************

[Investigator: For the convenience of the unit to verify my interview to you, please leave your name and contact number.]

Signature of interviewee ___________； phone number __________

Sign on behalf of the responder ______； phone number __________

This is the end of the investigation, thank you for your support and cooperation!

J Investigation Postscript

[Investigator: After the investigation, please continue to fill in the following questions according to the investigation situation]

J1 What kind of house does the old man live in now?

1 building 2 bungalows 3 adobe rooms 4 others (please specify) ____

J2 If it is a building, which floor do the elderly people live on? ______F

J3 If it's a building, is there an elevator?

1 Yes 0 No

J4 Were other people present during the investigation?

1 Yes 0 No

J5 Do other people present answer questions for you?

1 Yes 0 No [Jump to ask the question of J8]

J6 Answers on behalf of (the relationship with the elderly interviewees)

1 spouse 2 children 3 grandchildren 4 others (please specify) ______

J7 The reason for having someone answer the question instead :(multiple choice)

□Unable to answer because of deafness. □ Unable to answer because of dementia□ Inclear reply □ Hearing impairment□ Sick not available for visits □ Others (please specify) __________

J8 Health judgment of the elderly interviewed:

1 very healthy 2 relatively healthy 3 average 4 relatively unhealthy 5 very unhealthy

J9 Judgment on the self-care ability of the elderly interviewed:

1 completely self-care 2 partially self-care 3 completely unable to self-care
