# Supplementary material for: The silent epidemic: exploring the link between loneliness and chronic diseases in China’s elderly
Source: BMC Geriatr. 2024 Aug 26;24:710. doi: 10.1186/s12877-024-05163-2 (PMC11346041; doi:10.1186/s12877-024-05163-2)
Supplement: Supplementary file 4 — Supplementary Material 4 [file 12877_2024_5163_MOESM4_ESM.pdf]

The 4th Sample Survey of the Aged Population in Urban and Rural China (SSAPUR)

Directory of Investigator's Manual

The fourth sampling survey on the living conditions of the elderly in urban and rural China

Leading Group Office

Directory

A brief introduction to the fourth survey

II. Requirements for filling in the form

3. Index interpretation

4. Investigation steps

V. Investigator's Letter of Commitment

A brief introduction to the fourth survey

(1) Background investigation

Aging population is a basic national condition throughout the 21st century. By the end of 2014, China's elderly population aged 60 or above had reached 212 million, accounting for 15.5 percent of the total population. According to the prediction of the National Strategic Research Group of the Office of the National Working Commission on Aging, By 2015, China's population aged 60 and above will reach 221 million, and by 2053, it will peak at 487 million, accounting for 34.9 percent of the total population. The rapid development of aging population will have a long-term and profound impact on China's economic, political, cultural, social and ecological construction. The 18th National Congress of the Communist Party of China made the strategic deployment of "actively coping with the aging of the population". The National People's Congress clearly put forward that "actively dealing with the aging of the population is

a long-term strategic task of our country". To actively deal with the aging of the population needs scientific decision-making, and the first premise of scientific decision-making is to carry out comprehensive and in-depth investigation and research

"Sampling Survey on the Living Conditions of the Elderly in Urban and Rural China" is a sampling survey on the living conditions of the elderly conducted every five years under the leadership of the National Working Committee on Aging, sponsored by the National Office on Aging and approved by the National Bureau of Statistics. It is an important national condition survey in China. Since 2000, the National Office on Aging has carried out three programs, playing a positive role in understanding the basic living conditions of the elderly in both urban and rural areas of China and in formulating medium - and long-term development plans for undertakings for the aged as well as policies and regulations concerning the aged. At present, China's economic and social development has entered a new normal, and the aging of the population has entered a stage of rapid development. Many new changes have taken place in the living conditions of the elderly in urban and rural areas, and the aging work is faced with many new situations, new problems and new contradictions. Therefore, it is urgent to upgrade the level, expand the scope and enrich the content of the fourth survey.With the approval of State Councilor Wang Yong and the National Bureau of Statistics, The scope of the fourth survey was expanded from 20 provinces to 31 provinces (autonomous regions and municipalities directly under the central government) and Xinjiang Production and Construction Corps. The sample size was expanded from 20,000 to 224,000, and the survey content was further enriched.

(2) Significance of investigation

The fourth sample survey of the living conditions of the elderly in urban and rural China was jointly conducted by the National Office on Aging, the Ministry of Civil Affairs and the Ministry of Finance under the leadership of the National Working Committee on Aging. The fourth survey is an important survey of China's national conditions and an important measure taken by the central government to actively respond to the strategic deployment of an aging population. Good organization to carry out the investigation for the fourth time, to make clear

in the new period and pension service demand, China's urban and rural elderly living conditions for the party committees and governments at all levels of the CPC Central Committee and the State Council and to plan as a whole strategy to deal with an ageing population, planning and policy to provide scientific support, and to prepare the "aging" much starker choices-and graver consequences-in planning career development in China "to provide basic data support, it is of great significance.

(3) Investigation time

The fourth survey was conducted on August 1, 2015, and lasted for one month until August 31.

(4) Investigation objects

The fourth survey is a sampling survey, and the respondents are the elderly people aged 60 or above in mainland China who were selected during the survey period.

(5) contents of the investigation

The fourth survey focused on the living conditions of the elderly in urban and rural areas, focusing on family, health, care and nursing services, economy, social participation, rights protection, livable environment and spiritual and cultural life of the elderly.

II. Requirements for filling in the form

1. The questionnaire should be filled in with black pen.
2. All fillings should be written neatly and clearly, including the name, address of the cover, the description of the options and the text of the postscript of the investigator. Do not write in parallel or in traditional Chinese characters.
3. Please choose the answer according to the instructions on page 2 of the questionnaire.

Single choice: please tick one of the answers. Multiple choice: Please tick the box in □ before the appropriate choice. Be sure to ask about each item in multiple choice questions so that you don't miss out.

4. In the answers to some questions, we set up the answer "others (please explain) _________" because there is no way to exhaustive possible answers in advance. If investigators encounter such a situation, they should record the detailed answers of the elderly interviewed here.

5. "Survey Cover" to fill in: write down the survey location and the start time of the interview before the start of the questionnaire,

6. The investigator should let the interviewed old people (on behalf of the answer person) sign, and leave a telephone number before filling in the "survey postscript".

7. When the survey is over, fill in the closing time in Survey Cover in time.Fill in the Postscript to the Survey at the same time.

8. The investigators signed on the "Survey Cover" and left a contact phone number after self-examination of the questionnaire.

3. Index interpretation

Part of the A

A2: Date of birth refers to the year and month of birth of the elderly interviewed, filled in with Arabic numerals. Year is represented by four digits and month is represented by two digits

Investigators asked the elderly to provide ID cards, and then fill in according to the ID card. If the interviewed senior citizen does not have an ID card, please fill in according to the oral date of birth of the interviewed senior citizen or his/her family members.

A3: Household registration type, namely agricultural household registration, non-agricultural household registration. In areas where the distinction between agricultural and non-agricultural household registration has been cancelled, the household registration type should be "unified household registration". The surveyor chooses according to the type of household registration in the household registration book.

A4: Nationalities, except for the minorities listed in the options, other minorities should

indicate the specific name of the ethnic group. The contents in the household registration book or ID card shall prevail. Do not write the abbreviation when filling in the ethnic group, fill in the full name.

A5 : The term "uneducated" refers to a person who has never received school education at all levels and of all types provided by the State or other institutions running schools. This includes persons who have attended various literacy classes or adult literacy classes and have not subsequently attended any type of school education.

Primary education refers to the highest level of education received in primary school, whether in school, graduated, dropped out or not. Primary schools also included pre-liberation private schools.

Junior high school education refers to the highest level of education received by a person, whether in school, graduated, dropped out, or dropped out. Technical school equivalent to junior high level, also fill in this option.

High school education refers to the highest level of education received by a person, whether in school, graduated, drop-out or dropout, as a general high school, vocational high school, and secondary professional school. Technical school equivalent to high school level, also fill in this option.

The term "junior college" refers to the highest level of education received as a junior college. This option is filled in by those who are studying a college subject in an ordinary institution of higher learning, whether in school, graduate, drop-out or dropout.

Graduates who teach courses in accordance with the syllabus of college specialty issued by the Ministry of Education in correspondence universities, night universities and other universities held by radio and television universities, workers' universities and colleges authorized by the state to recognize their academic qualifications should fill in this option. Students who have not studied or are still in school shall fill in according to their previous educational level.

Those who have passed the national self-study examination and obtained a college diploma through self-study, should also fill in this option.

Bachelor's degree or above refers to the highest level of education received as a bachelor's degree or above. This is for undergraduate students who are enrolled in a regular institution of

higher learning, whether they are in school, have graduated, do not study or have dropped out.

Graduates who teach in accordance with the undergraduate syllabus issued by the Ministry of Education in correspondence universities, night universities and other forms of colleges and universities authorized by the state to recognize their academic qualifications should fill in this option. Students who have not studied or are still in school shall fill in according to their previous educational level.

This option is also filled in by those who have passed the examination and obtained a bachelor's degree through self-study and further study of university courses.

A6 Professional and technical titles refer to the professional and technical levels, abilities and achievements of professional and technical personnel, reflecting their technical levels and working abilities. Professional title is divided into senior, deputy senior, intermediate, assistant level (primary), technician level 5 levels.

A7 Independents have four characteristics: First, they do not belong to any political party and have non-partisan status; Second, it makes positive contributions to society and has certain social influence; Third, they have received good education and have high quality; Fourth, it has the identity of the object of united front work.

A8: Marital status

Have a spouse: The interviewees are married and have a spouse at the time of the survey

Death of a spouse: The elderly people interviewed once had a spouse, but the spouse had died at the time of the survey and had not remarried. If the elderly interviewees have lost their spouse for more than one time, they should fill in the time since the last time of losing their spouse.

Divorce: The elderly interviewed had spouses but were spousal free at the time of the survey. If the interviewed elderly people divorced more than once, then fill in the time since the last divorce

Part of the B

B1 "Children" refers to living biological children, adopted children and stepchildren, excluding daughter-in-law and son-in-law.

B1.5 Rotating support refers to the situation in which the elderly people interviewed have been living with two or more children in rotation by agreement (including verbal agreement) in the past 12 months.

B2 The term "living and eating together" refers to the current living state of living and eating together with the elderly interviewed. Although the registered permanent residence together, but for a long time (more than half a year) do not live together, can not be counted as the same as eating and living. Among them, nanny refers to eat and live in the home of the elderly domestic service personnel. Part-time work in the old people's homes only during the day does not belong to the situation of living with the same food. If the elderly are supported by their children in turn, the information of the families with their children living and eating together with the elderly interviewed at the time of the survey should be filled in.

B3.2 If the elderly person has more than one child living outside the province and different children visit the elderly person at home each year, the child with the most frequent visits will be selected.

B5 Looking after the house does not include housework.

Do household chores, including laundry, cooking, sweeping and other daily household tasks.

To look after grandchildren, including the care and education of children.

B6 Relatives/friends, excluding relatives/friends with whom they live and eat.

B7 Your family refers to the extended family of the elderly person, including the parents (including in-laws and parents-in-law), spouse, children and family members of the elderly person interviewed.

B9 Your family, refers to the family members who eat and live together. All subsequent questions concerning "your family" in this questionnaire shall be defined in accordance with this interpretation.

Part of the C

C2 If there is no such thing as regular drunkenness, how many times does the elderly drink every week?

C3 Morpheus quality, feel for standard with old people oneself.

C4 See well (including wearing glasses): First, ask the visual acuity status of the elderly interviewees without glasses. If the visual acuity is not very good, ask the visual acuity status after wearing glasses, and choose according to the visual acuity status after wearing glasses. If the elderly does not have glasses, don't ask, and choose according to the visual acuity status without wearing glasses.

C5 To hear well (including wearing hearing AIDS): First, ask the hearing condition of the elderly who do not wear hearing AIDS. If the hearing is not good, ask the hearing condition after wearing hearing AIDS, and choose according to the hearing condition after wearing hearing AIDS. If the elderly do not have hearing AIDS, don't ask, and choose according to the hearing condition without wearing hearing AIDS

C8 Exercise:指 All kinds of physical activities, such as walking, playing ball, boxing and practicing Qigong, which are carried out consciously for the purpose of fitness, do not include housework and farming.

C9 Health care products: A popular term for health food. Nourishes and tonics of a non-pharmaceutical nature that are taken for the purpose of improving health or preventing and treating diseases are collectively referred to as health care products. National standard GB16740-97 "health (functional) food general standard" Article 3.1 health food is defined as: "Health (functional) food is a kind of food, has the common characteristics of general food, can regulate the function of the human body, suitable for specific people to eat, but not for the purpose of treating diseases”.

C10 Physical examination：A Physical examination is distinguished from a series of examinations performed in a hospital under the condition of general illness. It refers to a Physical examination performed in the absence of illness or illness.

C11 Chronic diseases: The self-report of the interviewed elderly was the standard choice.

Diseases of the reproductive system: These include diseases of the male reproductive system and diseases of the female reproductive system. Among them, the common symptoms of male reproductive system diseases include abnormal urination, pyuria, abnormal urinary secretions, pain, lumps, sexual dysfunction and male infertility related to urinary diseases; Diseases of female reproductive system mainly include chlamydia, human papillomavirus,

genital herpes, vaginitis and so on.。

C12.5 Treated with traditional methods: Cupping, scraping, acupuncture and moxibustion, etc

C15 Admission to hospital: As a patient, the interviewed elderly lived in the hospital for at least 24 hours, and the outpatient service on the same day was not included.

C16 To see a doctor/Admission to hospital: It does not include going to the pharmacy to buy your own medication

C16.1 Self-expenses (not reimbursable): Expenses directly borne by individuals in the reimbursement process of medical treatment do not include reimbursement and expenses paid in personal medical insurance account and new rural cooperative medical care account.

C16.2 Children or other persons, other persons not including spouses.

C17 The term "out-of-pocket purchase of drugs" refers to the expenses paid for the purchase of drugs in addition to attending medical institutions or being hospitalized.

C18 The basic medical insurance for urban workers is one of the components of China's medical insurance system (basic medical insurance for urban workers, basic medical insurance for urban residents, and new rural cooperative medical care). It is a social insurance system established to compensate workers for economic losses caused by disease risks. Pass unit of choose and employ persons and individual pay cost, fund of insurance of build medical treatment, after GINSENg protects personnel to see a doctor to see a doctor to produce medical treatment charge, give certain economic compensation by organ of agency of medical treatment insurance, in order to avoid or reduce laborer because of sick, the economic risk that cure waits for place to bear.

The basic medical insurance for urban residents is an integral part of social medical insurance. It is mandatory and takes the government as the leading method, with residents (families) paying the main contribution, supplemented by appropriate government subsidies. According to the principle that pay cost standard and treatment level are consistent, the medical treatment insurance system that provides medical treatment expenditure for urban dweller.

New rural cooperative medical system refers to the government organized, guided and supported by the voluntary participation of farmers, individuals, collectivists and governments to raise funds, with major diseases as the main planning system for farmers medical mutual aid.

The fund is raised by means of individual payment, collective support and government funding.

Serious illness insurance for urban and rural residents reimburses the high medical expenses incurred by urban and rural residents who suffer from serious diseases. The aim is to solve the problem of "poverty caused by disease and return to poverty due to disease", which is strongly reflected by the masses, so that the vast majority of people will no longer be trapped in economic difficulties due to disease.

The large-sum medical insurance for employees refers to the medical expenses that exceed the maximum payment limit of the basic medical insurance pooling fund for the purpose of solving the serious illness or serious illness of the insured personnel. On the basis of the basic medical insurance, a large-sum medical insurance system has been established under the overall planning at the municipal level.

Medical treatment at public expense, point to the country to ensure national staff and implement, a social security system that provides medical treatment of certain expense account limit and preventive service to enjoy personnel by the regulation through the branch of medical treatment and sanitation.

C19 Commercial health insurance is an insurance that takes the body of the insured as the subject matter of the insurance and ensures the insured to obtain compensation for the direct expenses or indirect losses caused by injuries caused by diseases or accidents. It includes sickness insurance, medical insurance, income protection insurance, and long-term care insurance.

C20 The health status of the interviewed old people was selected as the standard.

Part of the D

D1 Have a meal: Meals, not including cooking

Getting dressed involves finding clothes (including underwear, coats, shoes) in closets and drawers and putting them on.

Go to the toilet, including unclothe, defecate, wash hands after using the toilet, dress, etc.

To take a bath, to stand under a shower, to sit in a bathtub to take a bath or tub bath or to scrub the upper and lower body.

Cooking involves preparing the ingredients, cook the meal and serve it to the table.

Daily shopping, including deciding what to buy and pay for it.

D3 Smart wearables, such as smart glasses, smart watches and smart bracelets, refer to the general term for the intelligent design of daily wearables and the development of wearable devices by using wearable technology.

D7 Meal assistance services refer to dining tables for the elderly, door-to-door cooking and meal assistance services for the elderly.

Bath service refers to the bath service provided for elderly people who have difficulty taking a bath.

Day care refers to the services provided by day care centres for the elderly in the community . Community day care center for the elderly refers to a place where day care services such as meal supply, personal care, health care and rehabilitation, recreation and recreation are provided for the semi-disabled elderly who are unable to take care of themselves completely and need some care in daily life. It is a new mode of community home care service for the semi-disabled elderly, which is "to receive care and participate in activities in the daytime, and to go home at night to enjoy family life".

Part of the E

E1.3 The work unit before retirement refers to the last work unit of the interviewed old people when they retire.

Three types of foreign-funded enterprises: There are three types of foreign-invested enterprises established within the territory of China: Sino-foreign equity joint ventures, Sino-foreign cooperative ventures and wholly foreign-owned ventures.

E2 Gainful employment: In the month before the survey, the interviewed elderly people actually engaged in various production, management and service activities in order to obtain wages, rewards in kind or business income, excluding voluntary labor and public welfare labor.

E3.1 Net income, which is equal to annual gross income minus operating costs. If the income of the elderly interviewed is shared with family members, the net income of the elderly interviewed needs to be estimated.

E4 Pension money refers to the current and fixed deposits of the surveyed elderly and their partners in the bank, excluding pocket money or cash on hand.

E5 Old-age pension (retirement pension):Attend agency institution basic endowment insurance, or town worker is basic endowment insurance, or endowment insurance of town dweller society, or the endowment insurance gold that place of endowment insurance of new-style rural society gets.

The basic endowment insurance for urban workers is an endowment insurance system that aims at ensuring the basic life after retirement for urban enterprise workers.

Soial endowment insurance for urban residents is an endowment insurance system designed to guarantee the basic life of the non-working people with urban household registration when they are old.

The new rural social pension insurance is an endowment insurance system aimed at ensuring the basic life of rural residents when they are old.

Occupational annuity refers to the supplementary old-age insurance system established by government organs and public institutions and their employees on the basis of participating in the basic old-age insurance of government organs and public institutions。

Enterprise annuity refers to a form of old-age insurance established by enterprises and their employees according to national policies and economic conditions on the basis of participating in the basic old-age insurance according to law, aiming at improving the living standard of employees after retirement and making an important supplement to the basic old-age insurance of the state.

Commercial endowment insurance, it is a kind of commercial insurance, it is insurance object with the life of the person or the body, be in insurant old age is retired or when protect period expires, by insurance company sets the form of a kind of insurance that pays annuities by the contract.

Pension service subsidy, point to a kind of service subsidy or subsidy that the government provides to the elderly with difficulties in providing for the aged such as the elderly with low income, living alone, loss of ability。

Care subsidies point to the government to be aimed at partial deformity old people, old age, the allowance that retires cadre take care of nurse to provide or allowance.

Old Age Allowance is a kind of welfare allowance given by the government for the elderly.

The minimum living allowance is a subsidy given by the government to families whose

per capita income is below the minimum living standard set by the local government .

Five insurance subsidies refers to the subsidy granted by the government to rural residents who meet the requirements of the "Regulations on the Work of Providing for the Five Guarantees in Rural Areas".

No rescue subventions means the aid subvention granted by the government to the elderly aged 60 years or above among urban residents who are unable to work, have no source of income, have no support or dependant, or whose support or dependant really has no support or dependant ability。

The subsidy for family planning families refers to the subsidy provided by the government to couples who reach the age of 60 in rural areas for family planning families with only one child or two girls.

The special subsidy for family planning refers to the subsidy provided by the government to the couples who have not given birth or adopted a child after the death, injury, illness or disability of their only child in urban and rural areas.

E7 National debt/bonds refer to the debenture certificates issued to investors by the government, financial institutions, industrial and commercial enterprises, etc. when they directly borrow money from the society and promise to pay the interest at a certain interest rate and repay the principal on the agreed terms。

Stock refers to the ownership certificate issued by the joint stock company, which is a kind of securities issued by the joint stock company to each shareholder as the holding certificate for raising funds and to obtain dividends and bonuses. Each share represents a basic unit of ownership in the business.

Funds refer to securities investment funds that raise funds through the public offering of fund shares. A financial product that is managed and operated by the fund custodian and managed by the fund manager for the benefit of the fund share holders and in the form of portfolio, which shares the interests and risks.

Precious metals include the physical objects such as gold and silver, as well as the electronic transaction investment with leverage and the paper gold, paper silver of the bank kind.

E8 Property right, point to the right that the owner of house property has according to

national law set place, namely the summation of the building each rights and interests, namely building owner is right of possession of this building property, use, income and the right of disposition. Including both large property rights, including small property rights.

E9 Renting public housing refers to housing with property rights owned by renting units or collectives.

The term "renting a private house" refers to renting a house owned by an individual

E10 Personal daily living expenses. If the interviewed old people live together with their children, all daily living expenses are paid by their children, and the estimate should be filled in.

Communication expenditure refers to the expenses paid by the surveyed elderly for the purchase of telecommunication services (including telephone fees and Internet fees) and postal services.

E13 Food expenditure, point to board expense or eat meal money, the grain that oneself produces in rural area, vegetable, fruit also should press general price calculation cost expenditure.

E14 Total household expenditure refers to the total of all actual household expenditure except borrowing expenditure, including consumption expenditure, purchase and construction expenditure, transfer expenditure, social security expenditure and other expenditure. Expenditure statistics are reported as the total value of the goods or services actually purchased, whether paid in a lump sum, in instalments, or on credit, as long as the goods or services have been consumed.

E15 Total family income refers to the sum of salary income, net operating income, income from production, transfer income, social security income and other income received by all family members who live and eat with the elderly interviewed during the survey period, excluding borrowing income. The statistical standard of income is accurate with the amount that produces actually, no matter income is to fill hair or send in advance, what should be investigated period to get only should be calculated according to the facts, do not make apportionment.

E17 Live off one's parents，A condition in which a viable adult depends on his or her parents without being "weaned". However, this does not apply to children who receive support from

their parents for reasons such as schooling, physical disability, poverty due to illness, etc.

E18 Economic status was selected according to the self-rating criteria of the elderly interviewed.

Part of the F

F1 The date of completion of a house refers to the time when the building to which the house belongs was completed. If the house has been renovated, fill in according to the year of renovation; If the house has been rebuilt, if the area of reconstruction is larger than the original area, the time of completion shall be filled in as the time of reconstruction; If the reconstruction area is less than the original area, the completion time shall be filled in with the completion time of the original house。

F2 The floor area of the house shall be filled in according to the floor area of the house.

If only the area is known, the following formula can be used for conversion:

Usable area (including the usable area of the extension) ÷0.7 = floor area

The following points should be noted when filling in this item:

Households living in hotels or rented houses shall fill in their housing area according to the actual situation of their current residence.

Living in the workplace of the household, housing area should fill in its living room floor area.

The gross floor area of each household living in the same house shall be one part of the area of the room used independently by each household plus the area for common use (including kitchen, toilet, hallway, balcony, etc.). For two households living in the same house, the gross floor area shall be calculated at half for each household. One third for each of the three households; For four or more households, and so on.

F3 A room for living alone: Refers to the situation where an elderly couple has a separate room for living purposes only, not including sharing the room with grandchildren。

F4 Tap water, also known as piped tap water,: Water is conveyed by pipes that are purified by utilities.Your own well in the yard does not count as running water.

Gas/Natural Gas/Biogas: Pipeline gas, gas canisters, etc. are not included.

The indoor toilet: Formal toilet in the house (may be pumped or otherwise).

Bath/shower facilities: Fixed bath (tub) or shower faucet and other bath facilities in the

house.

F5 Mobile phone for the elderly：Mobile phones with large keys, large fonts, large volume, and long standby for the elderly, usually without an operating system.

Smartphone: Like a personal computer, a mobile phone with an independent operating system, an independent running space where users can install software, games, navigation and other third-party programs, and can achieve wireless intervention through the mobile communication network.

Ordinary mobile phone: Mobile phones other than old people's phones and smart phones.

F6.2 A serious injury requires medical attention: A condition that requires medical treatment but does not require prolonged bed rest.

F11 If the community where the senior citizen is interviewed does not have the facilities listed, no choice is made.

Part of the G

G1 Participate in cultural and scientific promotion activities: An activity carried out as an organizer or promoter.

G2 Public welfare organization: Non-governmental social organizations that do not take profit maximization as the primary goal, but pursue social welfare as the main goal.

Folk/Folk Culture Organization: A folk organization specializing in the study of folk customs and culture.

G3 Family: Based on lineage of several generations or dozens of generations, a small range of family and clan can be used confusedly.

The clan: Often represented by a surname, they live together in communities. It can be composed of more than one family

Ancestor worship and grave sweeping activities in the name of individuals or families are not counted as clan/clan activities.

G5 Elderly people in difficulty: This includes both those with financial difficulties and those who are physically disabled or ill and need help.

Assistance: Unremunerated assistance does not include the provision of services as a babysitter or as an hourly worker.

G8 Community Affairs：Community construction, such as building roads and houses etc.

Part of the H

H2 Preferential Card for Senior Citizens (Card): A certificate of preferential treatment for the elderly issued by the government, which enables the elderly to enjoy preferential, preferential or reduced treatment in public services such as medical treatment and transportation.

H5 The situation experienced by the elderly interviewed does not include the situation that the elderly interviewed have seen or heard of others.

H6 Legal aid: Legal aid agencies established by the government organize lawyers for legal aid, providing free legal services for people with economic difficulties or special cases, including the elderly.

Part of the I

I4 Have any place: n fact, when the elderly are asked about their knowledge of the activity places, the elderly's self-report shall prevail. If there are the following activity places in the community, but the elderly answer "do not know", the investigator should choose "do not know".

I6 Travel: The elderly people who were interviewed left their usual living environment for leisure purposes, went to certain places and stayed there, but for no more than one year. If the interviewed elderly people plan to visit their children or relatives in other places, and their main purpose is to provide for the aged or take care of children, they will not be considered as traveling. But old people seasonally go to different places endowment calculates travel.

I9 Loneliness: In this survey, the term “loneliness” is defined as a one-dimensional emotional state based on the elderly participant's self-perception of lacking social connection or emotional support. The surveyor should only mark the "Often" term when the interviewee explicitly expresses a persistent state of loneliness.

I11 Mental age: A psychological term relative to natural age. Natural age is the objective age of an individual determined according to the number of years after birth. The mental age refers to the individual age determined according to the soundness of the individual's mental activities. Its main basis is : (1) The psychological process of cognition, emotion and will, which are unified with thinking and language as the core, developed by individuals in social practice; (2) Individuals constitute the unique psychological organization system of conscious activities.

4. Investigation steps

1. The investigator should contact the interviewed elderly in advance in strict accordance with the sampling list of the elderly roster in the selected community (village/residence) to determine the investigation time. If the selected elderly refuse to visit, die, migrate, cannot contact (contact more than three times), stay out for a long time, live in a nursing institution far away (outside the county (district)) and other circumstances, the selected elderly will be given up and re-selected in order from the alternative list.

2. Before entering the house, investigators should prepare the materials needed for the survey in advance, including the survey card, ID card, questionnaire, investigator manual, signature pen, mobile phone, etc., and fill in the cover of the questionnaire and other information.

3. After the investigator enters the house, he will read out the investigation guide first, fill in the first three items of Part A, and then officially start the investigation.

4. After the survey, the investigators should carefully review the questionnaire to ensure that there are no missing items and mistakes, and then sign and fill in the contact number.

5. After the completion of all questionnaires, they should be handed over to the township supervisor.

Investigator's Undertaking

_________ (Given name) _________ (Family name)，from____________________(unit), From August to September 2015, I participated in the fourth sample survey on the living conditions of the elderly in urban and rural China jointly carried out by the Office of the National Working Commission on Aging, the Ministry of Civil Affairs and the Ministry of Finance. With enthusiasm for the cause of the aged and a serious and responsible attitude towards the investigation work, I make voluntary commitment to:

1. Abide by the law and discipline, and comply with the relevant regulations and requirements of this investigation.

2. Never take advantage of the investigation opportunity to engage in any activities unrelated to the investigation.

3, Seek truth from facts, do not falsify, ensure that each questionnaire is true and effective.

4. Pay attention to the personal safety of yourself and your colleagues to avoid conflicts and accidents.

5. Pay attention to confidentiality and do not disclose the information of this investigation to others.

If you violate the above commitments, you are willing to accept the organization's handling and bear the civil liability for personal fault.

The signature：

The date； __(month) __(date) 2015(year)
